# Epidemiology of Diabetes Mellitus in Patients With Psoriatic Arthritis: Current Evidence

**DOI:** 10.1002/dmrr.70239

**Published:** 2026-10-27

**Authors:** Jiao Wang, Xuan Zhou, Jiale Chen, Qingyun Wang, Xiaofei Yi, Pengbo Gao, Jingjie He, Shufen Yang, Miao Zhang, Xiaoying Sun, Liu Liu, Xin Li

**Affiliations:** ^1^ Department of Dermatology Yueyang Hospital of Integrated Traditional Chinese and Western Medicine Shanghai University of Traditional Chinese Medicine Shanghai China; ^2^ Institute of Dermatology Shanghai Academy of Traditional Chinese Medicine Shanghai China

**Keywords:** diabetes mellitus, meta‐analysis, prevalence, psoriatic arthritis

## Abstract

**Aims:**

Diabetes mellitus (DM) is a common comorbidity of psoriatic arthritis (PsA); however, its global prevalence and the impact of treatment exposure remain unclear. This study aimed to assess the global prevalence of DM in patients with PsA using different subgroup analyses.

**Materials and Methods:**

A systematic search of PubMed, Embase, the Cochrane Library and the Web of Science was performed from inception until February 2025 for observational studies reporting the prevalence or incidence of DM in PsA patients. Random‐effects models were applied to pool estimates, and subgroup, meta‐regression, and sensitivity analyses were conducted to explore heterogeneity. Publication bias was evaluated using the Egger's test and the trim‐and‐fill method.

**Results:**

Sixty‐three studies, including 293,582 patients with PsA, were analysed. Patients with PsA included in this study were divided into treatment‐disclosed and non‐treatment‐disclosed groups. DM overall prevalence in patients with PsA was 13.84% in the non‐treatment‐disclosed population and 13.50% in the treatment‐disclosed cohort. Geographically, the comorbidity prevalence rates were higher in South America (37.98%) and Asia (16.76%) than in Europe (12.81%) and North America (12.38%) in the non‐treatment‐disclosed population. In the treatment‐disclosed population, prevalence was also highest in South America (28.13%), followed by Asia (18.30%). Meta‐regression analysis identified age, type of DM, region, and study design as contributors to heterogeneity. Evidence of small‐study effects was detected in the T2DM subgroup and cohorts receiving systemic therapy plus biologics.

**Conclusions:**

Subgroup analysis shows that the prevalence of DM is associated with the type of study and the region in which the patients were located. These findings support routine metabolic screening for PsA and highlight the need for longitudinal studies to track disease activity, treatment patterns, and metabolic outcomes.

**Abbreviations**
CI: Confidence interval
DM: Diabetes mellitus
MetS: Metabolic syndrome
MOOSE: Meta‐Analysis of Observational Studies in Epidemiology
PRISMA: Preferred Reporting Items for Systematic Reviews and Meta‐Analyses
PsA: Psoriatic arthritis
T1DM: Type 1 diabetes mellitus
T2DM: Type 2 diabetes mellitus

## 1 Introduction

Psoriatic arthritis (PsA) is a chronic systemic inflammatory arthropathy belonging to the seronegative spondyloarthropathy spectrum. It is typically negative for rheumatoid factor and anti‐cyclic citrullinated peptide antibodies and affects approximately 30% of patients with psoriasis [1]. PsA is characterised by peripheral arthritis, enthesitis, and axial spondyloarthritis, which typically coexist with psoriatic cutaneous/nail manifestations [2].

PsA is increasingly recognized as an independent disease rather than a comorbidity of psoriasis. The temporal relationship between PsA and cutaneous or nail psoriasis is variable, with skin or nail manifestations occurring either before or after the onset of arthritis [3]. Multi‐omics analyses have also revealed obvious differences between PsA and psoriasis [4].

PsA increases the risk of comorbidities, including metabolic syndrome (MetS) [5], osteoarthritis [6], and depression [7]. Diabetes mellitus (DM), a primary disease associated with MetS, has shown a potential relationship with PsA in previous research [8, 9]. DM is classified into type 1 diabetes mellitus (T1DM), type 2 diabetes mellitus (T2DM), gestational diabetes, and several hybrid or special forms [10]. Among these categories, T2DM represents over 90% of global DM cases [11].

PsA and DM share overlapping pathophysiological mechanisms, which may explain their frequent coexistence. This overlap has been consistently reported in both mechanistic and clinical studies. In PsA, chronic systemic inflammation is driven by cytokines such as TNF‐α and IL‐17 [12, 13]. These mediators promote joint damage and are implicated in insulin resistance and β‐cell dysfunction [14, 15]. Furthermore, alterations in the gut microbiome of patients with PsA may increase intestinal permeability and systemic inflammatory burden, thereby creating a metabolic milieu that favours insulin resistance [16, 17, 18, 19].

Additionally, PsA is commonly associated with dyslipidemia and lipid factor imbalance, characterised by elevated leptin and resistin levels [20, 21, 22, 23, 24]. These adipokines are recognized for their role in increasing systemic inflammatory responses and interfering with normal lipid metabolism and insulin signalling, thereby contributing to metabolic dysfunction in PsA [25]. A cross‐sectional study has shown that resistin levels are significantly higher in patients with PsA than in healthy individuals [26]. Resistin appears to promote inflammation by activating inflammatory pathways linked to the endocannabinoid system, while simultaneously weakening insulin action [27]. Clinical observations further suggest that elevated leptin levels are more frequently observed in female patients and individuals with obesity and are associated with an increased risk of T2DM in psoriasis cohorts [28], indicating possible sex‐specific pathways in PsA‐DM comorbidity.

Despite the growing body of literature on PsA metabolic comorbidities, the epidemiological characteristics of PsA–DM remain unclear. Furthermore, the reported prevalence estimates vary widely across studies, with the global prevalence of DM in patients with PsA being unclear. In addition, few studies have compared the outcomes according to the treatment, location, or subtype of DM. To address these questions, we systematically synthesised the available evidence through a meta‐analysis to assess and define the disease burden in patients with PsA.

## 2 Materials and Methods

This study was conducted in accordance with the Preferred Reporting Items for Systematic Reviews and Meta‐Analyses (PRISMA) 2020 statement (Table S1) and Meta‐Analysis of Observational Studies in Epidemiology (MOOSE) guidelines (Table S2).

### 2.1 Literature Search

A systematic literature search was conducted in the PubMed, Embase, the Cochrane Library, and Web of Science databases from inception to February 2025. The search protocols combined controlled vocabulary and entry terms. The main search terms were “psoriasis,” “diabetes mellitus,” “glucose intolerance,” “prediabetic state,” “scleredema adultorum,” “glycation end products,” “diabetic,” “incidence,” “prevalence” and “epidemiology.”

### 2.2 Inclusion and Exclusion Criteria

The inclusion criteria were as follows: (1) studies reporting quantifiable DM metrics (absolute numbers or percentages) to calculate the prevalence and/or incidence of DM in PsA; (2) observational studies, including cohort, case‐control, cross‐sectional, and real‐world studies; (3) studies using standardized diagnostic criteria for both PsA and DM; and (4) those with full texts available in English. The exclusion criteria were as follows: (1) duplicate studies; (2) studies employing nonobservational methodologies; (3) studies lacking extractable data for outcome assessment; (4) book chapters or other nonpeer‐reviewed sources; (5) letters, correspondence, commentaries, or other publication types that did not provide original data or did not meet the predefined eligibility criteria; (6) studies focusing solely on psoriasis without confirmed PsA; and (7) studies not written in English.

### 2.3 Data Extraction

Data were independently extracted by two investigators. The collected information included the first author, year of publication and study conduct, study design, country, diagnostic criteria for PsA and DM, sex distribution, DM subtypes, number of patients with PsA with comorbid DM, and details of therapies reported in the treatment‐disclosed studies.

### 2.4 Quality Assessment

Two reviewers independently evaluated the methodological quality of the included studies. For studies that did not explicitly report their study design, the same two reviewers independently classified the design to select the appropriate quality assessment tool. Cross‐sectional studies were assessed for the risk of bias using the Agency for Healthcare Research and Quality Checklist, whereas case–control and cohort studies were evaluated using the Newcastle–Ottawa Scale (NOS). According to standardized scoring thresholds, the study quality was classified as low (0–3 points), moderate (4–7 points), or high (8–11 points).

### 2.5 Data Analysis

Analysis was performed using R Studio, which primarily employs the meta and metafor packages. Given the anticipated between‐study heterogeneity across the observational studies, pooled prevalence estimates were calculated using the metaprop function in a random‐effects model. Heterogeneity was quantified using the I^2^ test. Subgroup, meta‐regression, and sensitivity analyses were performed to explore sources of heterogeneity. For subgroup analyses by study design, only studies that explicitly reported their study design were included. In the non‐treatment‐disclosed cohort, models were fitted using subgroup variables as predictors with the intercept suppressed (−1) so that each subgroup level corresponded to its own regression coefficient. Publication bias for subgroups within > 10 studies was detected using the Egger's test (Egger's linear regression test). When publication bias was detected, the trim‐and‐fill algorithm was applied to potentially missing studies and the effect estimates were adjusted appropriately.

## 3 Results

### 3.1 Characteristics of the Included Studies

The initial database search yielded 3135 relevant articles, of which 458 duplicate records were identified and excluded. The titles and abstracts of 2677 publications were screened. Of these, 510 met the preliminary eligibility criteria and completed the full‐text assessment. During the rigorous full‐text evaluation, 447 studies were excluded based on the preset exclusion criteria. Consequently, 63 studies were selected for the meta‐analysis. The complete screening workflow is detailed in Figure 1.

**FIGURE 1:**
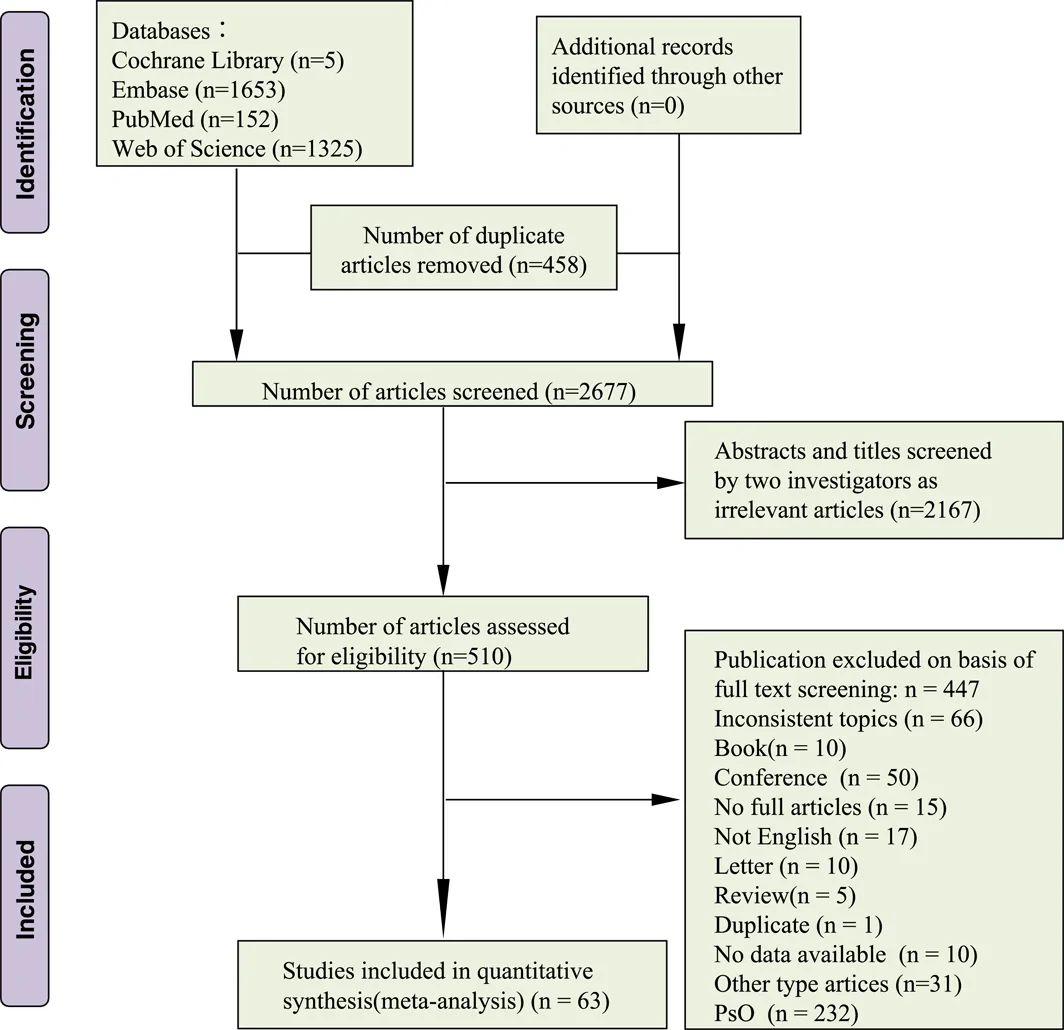
Flow diagram according to PRISMA 2020 guidelines.

This systematic review included 63 observational studies that investigated PsA‐DM comorbidity [8, 9, 29, 30, 31, 32, 33, 34, 35, 36, 37, 38, 39, 40, 41, 42, 43, 44, 45, 46, 47, 48, 49, 50, 51, 52, 53, 54, 55, 56, 57, 58, 59, 60, 61, 62, 63, 64, 65, 66, 67, 68, 69, 70, 71, 72, 73, 74, 75, 76, 77, 78, 79, 80, 81, 82, 83, 84, 85, 86, 87, 88, 89]. These articles were stratified by treatment disclosure status: 27 articles neither addressed PsA‐specific therapies nor included patients undergoing active treatment [29, 30, 31, 32, 33, 34, 35, 36, 37, 38, 39, 40, 41, 42, 43, 44, 45, 46, 47, 48, 49, 50, 51, 52, 53, 54, 55], whereas 36 included treatment‐disclosure cohorts [8, 9, 56, 57, 58, 59, 60, 61, 62, 63, 64, 65, 66, 67, 68, 69, 70, 71, 72, 73, 74, 75, 76, 77, 78, 79, 80, 81, 82, 83, 84, 85, 86, 87, 88, 89]. The following analyses were based on these two cohorts: non‐treatment‐treatment‐ and treatment‐disclosed cohorts. Among the non‐treatment‐disclosed studies (*n* = 27), two articles included both children and adults [35, 40], 14 included adults only [29, 30, 32, 33, 34, 36, 37, 38, 41, 42, 43, 52, 53, 55], and 11 did not mention the patient's age [31, 39, 44, 45, 46, 47, 48, 49, 50, 51, 54]. Four studies defined their cohorts as patients with T2DM [33, 36, 38, 54], whereas others provided no diabetic classification [29, 30, 31, 32, 34, 35, 37, 39, 40, 41, 42, 43, 44, 45, 46, 47, 48, 49, 50, 51, 52, 53, 55]. Three articles revealed significant gender disparities in both PsA prevalence and DM comorbidity [30, 34, 43]. The breakdown included eight cross‐sectional studies [34, 37, 38, 39, 43, 44, 48, 54], two case‐control studies [49, 51] and seven cohort studies [29, 30, 33, 41, 42, 45, 55]. The treatment‐disclosed group encompassed 16 cross‐sectional [8, 9, 61, 64, 67, 72, 75, 76, 77, 79, 80, 81, 85, 87, 88, 89], one case‐control [57], and six cohort studies [56, 59, 62, 66, 68, 74], demonstrating two mixed‐age cohort [63, 83], 20 adult‐specific investigations [9, 57, 60, 62, 64, 68, 69, 70, 72, 73, 74, 75, 76, 77, 80, 81, 82, 85, 88, 89], and 14 studies lacking age documentation [8, 56, 58, 59, 61, 65, 66, 67, 71, 78, 79, 84, 86, 87]. Treatment‐stratified analysis identified differential efficacy patterns. The detailed characteristics of the included studies are presented in Tables S3 and S4.

### 3.2 Quality of Included Studies

For the cross‐sectional studies, 21 treatment‐disclosed group studies were assessed: four were of high quality [8, 9, 76, 87], 17 of moderate quality [61, 63, 64, 67, 69, 71, 72, 75, 77, 78, 79, 80, 81, 85, 86, 88, 89], and none were classified as low quality. In the non‐treatment‐disclosed group (16 studies), three were of high quality [35, 46, 54] and 13 of moderate quality [31, 32, 34, 37, 38, 39, 40, 43, 44, 47, 48, 52, 78], with no study rated as low quality. For the cohort studies, among the treatment‐disclosed group (12 studies), five were of high quality [59, 66, 68, 82, 84] and seven of moderate quality [56, 58, 60, 62, 70, 74, 83], while in the non‐treatment‐disclosed group (eight studies), three were of high quality [33, 45, 55] and five of moderate quality [29, 30, 36, 41, 42]; again, no study was categorised as low quality. For the case‐control studies, two treatment‐disclosed group studies were available, both of which were of moderate quality [57, 65]. In the non‐treatment‐disclosed group (three studies), one was of high quality [51], and two were of moderate quality [49, 50]. The detailed results of the quality assessment are presented in Tables [Link], [Link], [Link], [Link].

### 3.3 Outcomes of Non‐treatment‐disclosed Studies

#### 3.3.1 Subgroup Analysis of Prevalence

The meta‐analysis results revealed an aggregate PsA‐DM comorbidity prevalence of 13.84% (95% confidence interval (CI): 10.72%–17.69%; random‐effects model; *p* < 0.00005, Table 1, Supporting Information S2: Figure S1A) with significant heterogeneity between studies.

**TABLE 1:** Meta‐analysis of the prevalence of DM in non‐treatment‐disclosed patients with PsA.

Subgroup	Non‐treatment‐disclosed PsA‐DM							
	Study (*n*)	Prevalence [95% CI]	I^2^	*p* value for heterogeneity	Egger value	Trim value	Meta regression Coef. [95% CI]	*p* value for meta‐regression
Overall prevalence	27	13.84 [10.72; 17.69]	98.9%	*p* < 0.00005	*p* = 0.87	12.91 [9.91; 16.66]	—	—
Age group								
Adults + Children	2	13.54 [3.96; 37.30]	97.7%	*p* < 0.0001	—	—	−1.85 [−2.89; −0.80]	*p* = 0.0005
Adults	25	14.76 [11.70; 18.46]	99%	*p* < 0.00005	*p* = 0.74	13.14 [10.19; 16.80]	−1.74 [−2.03; −1.46]	*p* < 0.0001
DM type								
T2DM	4	7.16 [5.18; 9.82]	96.9%	*p* < 0.0001	—	—	−2.56 [−3.16; −1.96]	*p* < 0.0001
DM	21	16.76 [13.36; 20.81]	99%	*p* < 0.00005	*p* = 0.47	14.15 [10.89; 18.18]	−1.59 [−1.86; −1.33]	*p* < 0.0001
Region								
Asia	6	16.76 [11.82; 23.22]	86.3%	*p* < 0.0001	—	—	−1.59 [−2.11; −1.07]	*p* < 0.0001
Europe	10	12.81 [8.22; 19.43]	99.5%	*p* < 0.00005	*p* = 0.49	17.95 [10.84; 29.72]	−1.91 [−2.31; −1.51]	*p* < 0.0001
North America	7	12.38 [10.05; 15.17]	98.8%	*p* < 0.0001	—	—	−1.94 [−2.38; −1.50]	*p* < 0.0001
South America	2	37.98 [30.03; 46.64]	0%	*p* = 0.4530	—	—	−0.49 [−1.42; 0.44]	*p* = 0.3034
Study design								
Cohort study	7	11.61 [7.71; 17.49]	99.4%	*p* < 0.0001	—	—	−2.15 [−2.59; −1.71]	*p* < 0.0001
Cross‐sectional study	8	15.42 [9.63; 24.71]	94.9%	*p* < 0.0001	—	—	−1.87 [−2.28; −1.45]	*p* < 0.0001
Case‐control study	2	27.87 [26.79; 29.00]	0%	*p* = 1.0000	—	—	−1.28 [−2.08; −0.47]	*p* = 0.0019

##### 3.3.1.1 Subgroup Analysis of Age

In this study, individuals aged > 17 years were defined as adults. Age‐stratified analyses revealed that comorbidity prevalence was 14.76% in adults (95% CI: 11.70%–18.46%; random‐effects model; *p* < 0.00005, Table 1, Supporting Information S2: Figure S1B). Owing to the limited number of paediatric studies, subsequent subgroup analyses concentrated on adult cohorts to reduce potential bias.

##### 3.3.1.2 Subgroup Analysis of the Type of DM

DM can be classified into T1DM or T2DM. However, some articles did not specify the type of DM. Therefore, the analysis was mainly performed on articles classified into the DM type subgroup. The prevalence of T2DM was 7.16% (95% CI: 5.18%–9.82% random‐effects model; *p* < 0.0001) and unclassified DM was 16.76% (95% CI: 13.36%–20.81%; random‐effects model; *p* < 0.00005, Table 1, Supporting Information S2: Figure S1C).

##### 3.3.1.3 Subgroup Analysis of the Region

These articles include data from four continents: Europe, Asia, North America, and South America. South America showed the highest prevalence (37.98% [95% CI: 30.03%–46.64%; random‐effects model; *p* = 0.4530]), followed by Asia (16.76% [95% CI: 11.82%–23.22%; random‐effects model; *p* < 0.0001]), Europe (12.81% [95% CI: 8.22%–19.43%; random‐effects model; *p* < 0.00005]) and North America (12.38% [95% CI: 10.05%–15.17%; random‐effects model; *p* < 0.0001]). Analysing the prevalence in different countries, it was found that Brazil (41.27%), Mexico (34.85%), and Italy (33.33%) had the highest prevalence; this was followed by Saudi Arabia (30.16%), Israel (23.52%), the Slovak Republic (19.21%); the lowest prevalence rates were found in Belgium (6.49%). The global prevalence of psoriasis and DM comorbidities is shown in Table 1, Figure 2 and Supporting Information S2: Figure S1D‐S1F.

**FIGURE 2:**
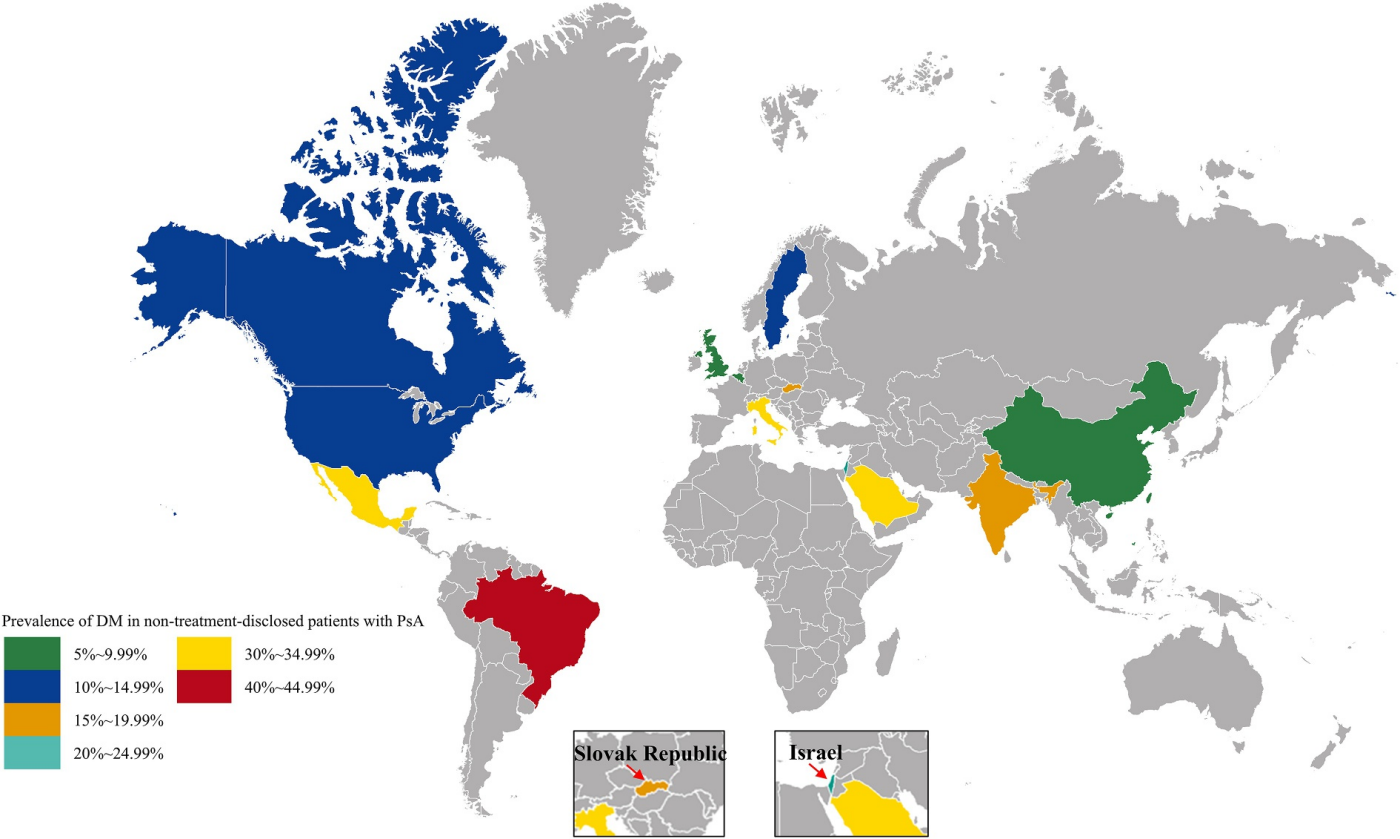
Prevalence of DM in non‐treatment‐disclosed patients with PsA in different countries.

##### 3.3.1.4 Subgroup Analysis of Study Design

This meta‐analysis included three types of studies: seven cohort studies, eight cross‐sectional, and two case‐control studies. Analyses by study type showed that the prevalence of PsA and DM comorbidities was highest in case‐control studies (27.87% [95% CI: 26.79%–29.00%; random‐effects model; *p* = 1.000]), followed by cross‐sectional studies (15.42% [95% CI: 9.63%–24.71%; random‐effects model; *p* < 0.0001]) and cohort studies (11.61% [95% CI: 7.71%–17.49%; random‐effects model; *p* < 0.0001]) (Table 1, Supporting Information S2: Figure S1G).

#### 3.3.2 Meta‐Regression Analysis

Meta‐regression analyses were performed to explore the sources of heterogeneity in the non‐treatment‐disclosed cohorts. The results indicated that the age group (*p* = 0.0005 for adults + children, *p* < 0.0001 for adults, and *p* = 0.0002 for children), DM type (*p* < 0.0001 for T2DM and *p* < 0.0001 for unspecified DM), region (*p* < 0.0001 for Asia, Europe, and North America; *p* = 0.303 for South America), and study design (*p* < 0.0001 for cohort and cross‐sectional studies; *p* = 0.0019 for case‐control studies) were significant contributors (Table 1). These findings suggest that demographic composition, disease classification, geographic distribution, and methodological design may account for a substantial proportion of the between‐study heterogeneity. However, given the extremely high I^2^ values across analyses, the explanatory power of these covariates should be interpreted with caution.

#### 3.3.3 Sensitivity Analyses

Sensitivity analyses yielded consistent results after excluding individual trials, indicating the robustness of the analytical model (Table 1, Supporting Information S2: Figure S1H‐S1K).

#### 3.3.4 Publication Bias

Potential publication bias in the non‐treatment‐disclosed cohort was evaluated using Egger's linear test. The overall Egger's test did not indicate a significant small‐study effect (*p* = 0.873). Similarly, subgroup analyses did not reveal a consistently biased signal in the adult, European, or unclassified DM subgroups. Trim‐and‐fill analysis yielded an adjusted pooled prevalence of 12.91% (95% CI: 9.91%–16.66%). These findings suggest that the pooled estimates in the non‐treatment‐disclosed populations are unlikely to be substantially affected by publication bias (Table 1, Supporting Information S2: Figure S2A‐S2L).

### 3.4 Outcomes of Treatment‐Disclosed Studies

#### 3.4.1 Subgroup Analysis of Prevalence

The meta‐analysis results for treatment‐disclosed studies demonstrated an overall PsA‐DM comorbidity prevalence of 13.50% (95% CI: 11.28%–15.90%; random‐effects model; *p* < 0.00005, Table 2 Supporting Information S2: Figure S3A).

**TABLE 2:** Meta‐analysis of the prevalence of DM in treatment‐disclosed patients with PsA.

Subgroup	Treatment‐disclosed PsA‐DM							
	Study (n)	Prevalence [95% CI]	I^2^	*p* value for heterogeneity	Egger value	Trim value	Meta regression Coef. [95% CI]	*p* value for meta‐regression
Overall prevalence	36	13.50 [11.28; 15.90]	99%	*p* < 0.00005	*p* = 0.0965	17.11 [14.35; 20.07]	—	—
Age group								
Adults + children	2	10.10 [8.79; 11.59]	76.3%	*p* = 0.0399	—	—	−2.46 [−3.28; −1.65]	*p* < 0.0001
Adults	34	14.23 [12.28; 16.44]	98.6%	*p* < 0.00005	*p* = 0.1811	17.86 [15.24; 20.81]	−1.78 [−1.95; −1.61]	*p* < 0.0001
DM type								
T1DM	2	1.06 [0.23; 2.52]	78.5%	*p* = 0.0311	—	—	0.10 [−0.04; 0.24]	*p* = 0.1511
T2DM	9	13.47 [10.73; 16.46]	95.3%	*p* < 0.0001	*p* = 0.0071	19.68 [15.45; 23.91]	0.37 [0.32; 0.43]	*p* < 0.0001
DM	25	15.28 [12.59; 18.17]	98.6%	*p* < 0.00005	*p* = 0.9351	16.10 [13.30; 19.35]	0.40 [0.37; 0.44]	*p* < 0.0001
Region								
Europe	12	11.50 [10.29; 12.84]	64.3%	*p* = 0.0003	*p* = 0.4709	11.39 [10.03; 12.82]	−2.04 [−2.30; −1.78]	*p* < 0.0001
North America	10	13.21 [9.70; 17.75]	97.8%	*p* < 0.0001	*p* = 0.1253	19.72 [14.16; 25.95]	−1.86 [−2.14; −1.58]	*p* < 0.0001
Oceania	1	12.86 [10.02; 16.15]	—	—	—	—	−1.91 [−2.88; −0.95]	*p* = 0.0001
South America	2	28.13 [24.16; 32.47]	2.7%	*p* = 0.3107	—	—	−0.97 [−1.66; −0.28]	*p* = 0.0059
Asia	9	18.30 [14.08; 23.43]	81.7%	*p* < 0.0001	*p* = 0.6227	21.44 [16.30; 27.05]	−1.46 [−1.76; −1.16]	*p* < 0.0001
Study design								
Cohort study	6	13.92 [8.39; 23.10]	96.7%	*p* < 0.0001	—	—	−1.94 [−2.30; −1.59]	*p* < 0.0001
Cross‐sectional study	16	13.07 [11.06; 15.45]	92.7%	*p* < 0.0001	*p* = 0.9426	13.15 [10.92; 15.57]	−2.04 [−2.25; −1.84]	*p* < 0.0001
Case‐control study	1	12.08 [7.97; 17.31]	—	—	—	—	−2.11 [−3.10; −1.13]	*p* < 0.0001
Therapy								
Systemic therapy	1	10.41 [6.28; 15.95]	—	—	—	—	−2.15 [−3.36; −0.94]	*p* = 0.0005
Systemic therapy + biologics	22	14.39 [11.58; 17.75]	96.2%	*p* < 0.0001	*p* = 0.0349	20.42 [16.06; 25.61]	−1.76 [−1.99; −1.53]	*p* < 0.0001
Systemic therapy + biologics + phototherapy	1	20.43 [18.94; 21.98]	—	—	—	—	−1.36 [−2.47; −0.25]	*p* = 0.0166
Topical therapy + systemic therapy	2	11.63 [10.99; 12.30]	4.4%	*p* = 0.3515	—	—	−2.01 [−2.69; −1.34]	*p* < 0.0001
Topical therapy + systemic therapy + biologics	4	12.51 [10.16; 15.31]	63.7%	*p* = 0.0074	—	—	−1.92 [−2.35; −1.49]	*p* < 0.0001
Topical therapy + systemic therapy + biologics + phototherapy	2	19.12 [8.93; 36.29]	98.6%	*p* < 0.0001	—	—	−1.44 [−2.24; −0.64]	*p* = 0.0004

##### 3.4.1.1 Subgroup Analysis of Age

In age‐stratified analyses, adults exhibited a prevalence of 14.23% (95% CI: 12.28%–16.44%; random‐effects model; *p* < 0.00005, Table 2, Supporting Information S2: Figure S3B). However, in the statistics of two articles that included both children and adults, the prevalence decreased to 10.10% (95% CI: 8.79%–11.59%; random‐effects model; *p* = 0.0399, Table 2, Supporting Information S2: Figure S3B). Because of the scarcity of paediatric studies, subsequent subgroup analyses were conducted exclusively in adults to ensure the reliability of the findings.

##### 3.4.1.2 Subgroup Analysis of the Type of DM

In studies that specified DM subtypes, T2DM prevalence was reported as 13.47% (95% CI: 10.73%–16.46%; random‐effects model; *p* < 0.0001). In contrast, T1DM prevalence was considerably lower, at 1.06% (95% CI: 0.23%–2.52%; random‐effects model; *p* = 0.0311; Table 2, Supporting Information S2: Figure S3C).

##### 3.4.1.3 Subgroup Analysis of the Region

The articles covered five continents (Europe, Asia, North America, South America, and Oceania). South America showed the highest prevalence at 28.13% (95% CI: 24.16%–32.47%; random‐effects model; *p* = 0.3107), followed by Asia (18.30% [95% CI: 14.08%–23.43%; random‐effects model; *p* < 0.0001]), North America (13.21% [95% CI: 9.70%–17.75%; random‐effects model; *p* < 0.0001]), Oceania (12.86% [95% CI: 10.02%–16.15%), and Europe (11.50% [95% CI: 10.29%–12.84%; random‐effects model; *p* = 0.0003]). Country‐level analyses indicated the highest prevalence in Malaysia (29.03%), Brazil (29.01%), Greece (20.00%), America (18.23%) and China (17.71%), with lower rates in Turkey (14.63%), Spain (13.45%), Australia (12.86%), Israel (12.75%), France (12.12%), Denmark (11.57%) and Italy (10.61%). The lowest prevalence rates were observed in Canada (9.75%), Belgium (8.11%), and Poland (7.87%). Detailed visualizations are provided in Figure 3, Table 2, and Supporting Information S2: Figure S3D‐S3F.

**FIGURE 3:**
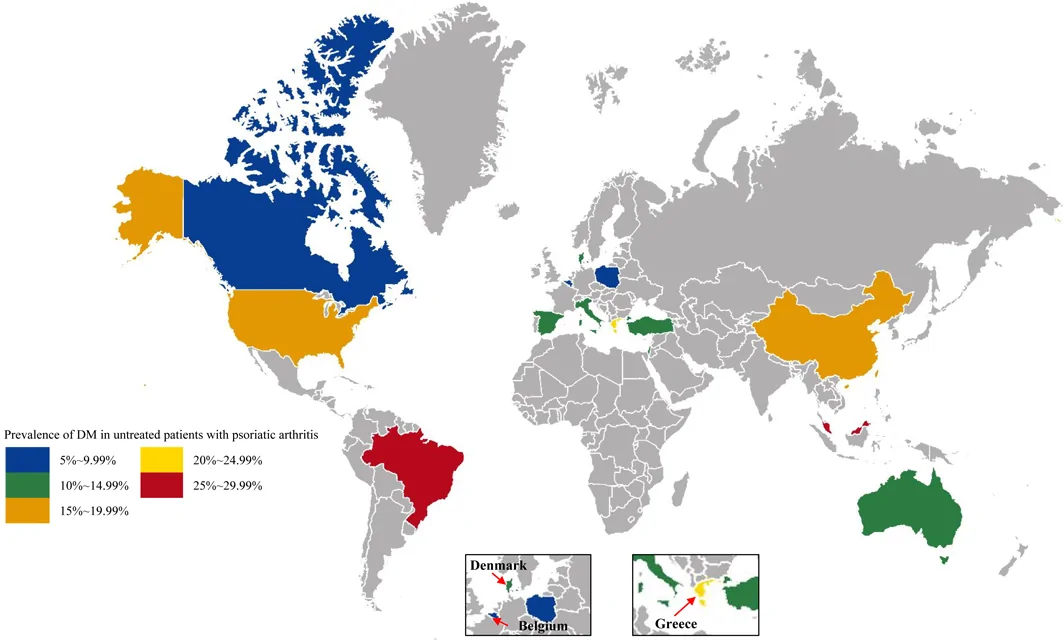
Prevalence of DM in treatment‐disclosed patients with PsA in different countries.

##### 3.4.1.4 Subgroup Analysis of Study Design

Three study types were analysed: case–control studies (*n* = 1), cohort studies (*n* = 6), and cross‐sectional studies (*n* = 16). Analyses by study type showed that the prevalence of PsA and DM comorbidities was highest in cohort studies (13.92% [95% CI: 8.39%–23.10%; random‐effects model; *p* < 0.0001]), followed by cross‐sectional studies (13.07% [95% CI: 11.06%–15.45%; random‐effects model; *p* < 0.0001]). The case‐control study reported a prevalence of 12.08% (95% CI: 7.97–17.31) (Table 2, Supporting Information S2: Figure S3G).

##### 3.4.1.5 Subgroup Analysis of Therapy

When stratified by therapeutic regimen, the pooled prevalence of DM among patients with PsA varied considerably. For systemic therapy alone, the prevalence was 10.41% (95% CI: 6.28%–15.95%) based on a single eligible study. Patients treated with systemic therapy combined with biologics had a prevalence of 14.39% (95% CI: 11.58%–17.75%; random‐effects model, *p* < 0.0001). The combination of systemic therapy, biologics, and phototherapy yielded the highest prevalence of 20.43% (95% CI: 18.94%–21.98%). Intermediate prevalence estimates were observed in regimens involving topical therapy with systemic therapy (11.63% [95% CI: 10.99%–12.30%; random‐effects model; *p* = 0.3515]) and in combinations including topical therapy, systemic therapy, and biologics (12.51% [95% CI: 10.16%–15.31%; random‐effects model; *p* = 0.0074]). For the subgroup receiving topical therapy, systemic therapy, biologics, and phototherapy, the prevalence was 19.12% (95% CI: 8.93%–36.3%; random‐effects model; *p* < 0.0001) (Table 2, Supporting Information S2: Figure S3H). Although regimens incorporating phototherapy showed relatively high prevalence estimates, these results should be interpreted cautiously because of the small number of included studies and, where estimable, substantial heterogeneity.

#### 3.4.2 Meta‐Regression Analysis

Meta‐regression analyses were performed to identify the sources of heterogeneity. The analyses revealed that age (*p* < 0.0001), type of DM (T2DM, *p* < 0.0001), region (Europe, North America, Asia, South America, and Oceania, all *p* < 0.0059), study design (*p* < 0.0001), and therapy mode (systemic therapy, systemic therapy combined with biologics, and combined regimens including phototherapy, all *p* < 0.0166) were significant contributors (Table 2). These findings suggest that demographic characteristics, disease classification, geographic distribution, methodological design, and therapeutic strategies were the primary sources of heterogeneity. However, given the extremely high I^2^ values across analyses, these results should be interpreted with caution.

#### 3.4.3 Sensitivity Analyses

Sensitivity analyses yielded consistent results after excluding individual trials, indicating the robustness of the analytical model (Supporting Information S2: Figure S4A‐S4I).

#### 3.4.4 Publication Bias

In the treatment‐disclosed population, Egger's test was performed for the overall analysis and for each subgroup. The overall Egger's test results did not reach significance (*p* = 0.0965). In the DM‐type subgroup, the T2DM group showed clear evidence of small‐study effects (*p* = 0.0071) and the trim‐and‐fill method suggested that the adjusted prevalence was higher than the original estimate, implying a potential underestimation. Within the therapy subgroups, only the “systemic therapy + biologics” group underwent Egger's test (*p* = 0.0349), indicating a certain degree of publication bias; other therapy subgroups with fewer than 10 studies were not assessed. Overall, although some subgroups demonstrated signs of publication bias, most results did not suggest a systematic bias. Given the extremely high heterogeneity (I^2^ = 99%), these findings should be interpreted with caution (Supporting Information S2: Figure S5A‐S5AA).

## 4 Discussion

This study estimated the global prevalence of DM in patients with PsA based on 63 observational studies involving 293,582 individuals. To reduce the potential impact of treatment‐induced DM, the included studies were divided into non‐treatment‐disclosed and treatment‐based groups based on whether the participants received PsA–specific pharmacological therapy [90, 91]. Contrary to initial expectations, DM prevalence was comparable between the two groups (13.84% vs. 13.50%). However, since all of the included studies were observational and treatment regimens were heterogeneous, this comparison should not be interpreted as evidence for the lack of a causal relationship between psoriasis treatment and the risk of diabetes.

When compared with earlier epidemiological work, our results align with a 2013 meta‐analysis that identified T2DM as a risk factor for PsA (odds ratio (OR) 2.18; 95% CI 1.36–3.50) [92]. A meta‐analysis of prospective cohort studies also showed that the risk of T2DM was 38%, higher in patients with PsA compared to non‐rheumatic populations (HR = 1.38, 95% CI:1.31–1.47) [93]. These findings support DM as a common clinical comorbidity of PsA. However, previous studies were limited by insufficient reporting of PsA‐specific treatment exposure and a lack of differentiation between T1DM and T2DM. In addition, most available data were derived from cohorts in Europe and North America, constraining the generalisability of their conclusions. The analysis included studies from a broad range of geographic regions. Subgroup analysis was performed according to the DM subtype and treatment disclosure, improving both the generalisability and specificity of the findings.

Substantial variations have been observed in the prevalence of DM across geographic regions. South America had the highest prevalence, although this was based on only two studies. Such regional differences may partly reflect variations in baseline metabolic risk among populations of different countries [94, 95]. The country‐level estimates suggest a relatively higher DM prevalence in Brazil and Malaysia, and lower DM prevalence in countries such as Poland. For most countries, prevalence estimates were derived from only 1 or 2 studies, making the apparent cross‐country variation largely exploratory rather than confirmatory. Therefore, the observed heterogeneity is more likely to reflect study‐level differences than the true population‐level epidemiology.

The study design also affected the prevalence of DM in patients with PsA. The pooled prevalence differed by study design, although the pattern was not consistent between the two groups. Cohort designs may capture comorbidities more comprehensively, whereas cross‐sectional studies provide a one‐time snapshot, which may underrepresent long‐term outcomes. In addition, the study design may have introduced selection bias. For instance, cohorts often originate from specialty clinics or healthcare databases, where patients may have more severe disease or longer disease duration, both of which could increase the risk of DM. In contrast, cross‐sectional studies may include a broader spectrum of disease severities at a single time point.

Our analysis suggests that the association between PsA and DM is associated with shared inflammatory and metabolic processes. Chronic systemic inflammation is a biological link between PsA and DM [96]. This link involves upregulation of pro‐inflammatory cytokines such as TNF‐α [97] and IL‐17 [98]. TNF‐α induces insulin resistance by activating the MEKK1‐MEK1/2‐ERK1/2 pathway, thereby inhibiting serine phosphorylation of IRS‐1 and impairing insulin signalling [99]. These pro‐inflammatory cytokines have also been shown to promote apoptosis of pancreatic β‐cells [100, 101]. Beyond inflammatory signals, the higher obesity rate in patients with PsA further amplifies the metabolic risk. Obesity and sedentary behaviour may reinforce the cycle of inflammation and metabolic abnormalities by inducing adipose tissue inflammation and exacerbating adipokine imbalance. This interaction worsens PsA symptoms and increases the incidence of T2DM [102].

This meta‐analysis had some limitations. Some included articles did not specify the subtype of the retrospective study. In addition, many studies did not clearly distinguish between T1DM and T2DM, which may have reduced the etiological specificity of the pooled estimates. Furthermore, few studies have provided stratification according to disease severity, duration, or treatment response, constraining a more nuanced assessment of metabolic heterogeneity within PsA populations. Finally, inconsistencies in reporting the temporal sequence between DM onset and PsA treatment initiation may partly underlie the absence of significant differences between the treatment‐disclosed and non‐treatment‐disclosed cohorts.

Our analysis yielded significant clinical insights despite certain limitations. It distinguishes the effects of PsA treatment from the prevalence of DM. This clarity helps better understand the risk and identify patients who might need more frequent metabolic monitoring. Finally, our results pave the way for further research to determine how things work and to develop new ways to eliminate and treat diseases.

## 5 Conclusion

This meta‐analysis is the first to provide a comprehensive global estimate of the prevalence of DM in patients with PsA across different treatment disclosures and regions. This meta‐analysis shows that approximately 14% of patients with PsA worldwide have DM, especially T2DM. Similar prevalence was observed in treatment‐disclosed and non‐treatment‐disclosed groups. Marked geographic variations are also identified, underscoring the complex DM risk profile of patients with PsA. Clinically, these results support routine metabolic screening for PsA management. Future longitudinal studies should monitor the disease activity, treatment patterns, and metabolic outcomes.

## Author Contributions

J.W. and X.Z. wrote the main manuscript text and performed formal analysis, data curation, investigation, and visualization. J.C. contributed to data curation, investigation, formal analysis, and visualization. Q.W. performed data curation and investigation and provided supervision. X.Y. contributed to data curation, methodology, and investigation. P.G. contributed to data curation, methodology, and visualization. J.H. and S.Y. performed data curation and investigation. M.Z. contributed resources and investigation. X.S. contributed to the investigation and revised the manuscript. L.L. contributed to conceptualization, methodology, supervision, and manuscript revision. X.L. contributed to conceptualization, methodology, supervision, funding acquisition, and manuscript revision. All authors reviewed and approved the final manuscript.

## Funding

This work was supported by the National Key Research and Development Programme (No. 2024YFC3505301), the National Natural Science Foundation of China (No: 82374445, 82675767), Shanghai Hospital Development Centre Foundation (No. SHDC12025132), CACMS Innovation Fund (No. CI2023C018LH), Shanghai Oriental Talent Programme for Top‐notch Project (No. BJWS2024046), 2025 Shanghai Municipal Health Commission Seed Program for Medical New Technology Research and Translation (No. 2025ZZ1010), Clinical Cohort Shanghai (No. SHDC25CCsS013).

## Ethics Statement

The authors have nothing to report.

## Consent

The authors have nothing to report.

## Conflicts of Interest

The authors declare no conflicts of interest.

## Supporting information


Supporting Information S1

Supporting Information S2

**Table S1:** PRISMA Checklist.

**Table S2:** Moose checklist.

**Table S3:** Characteristics of the included studies without treatment disclosure.

**Table S4:** Characteristics of the included studies with treatment disclosure.

**Table S5:** Risk of bias of the treatment‐disclosed cross‐sectional studies assessed using the Agency for Healthcare Research and Quality tool.

**Table S6:** Quality assessment of the treatment‐disclosed cohort and case‐control studies using the Newcastle‐Ottawa Scale.

**Table S7:** Risk of bias of the non‐treatment‐disclosed cross‐sectional studies assessed using the Agency for Healthcare Research and Quality tool.

**Table S8:** Quality assessment of the non‐treatment‐disclosed cohort and case‐control studies using the Newcastle‐Ottawa Scale.

## Data Availability

The data that support the findings of this study are available from the corresponding author upon reasonable request.

## References

[1] F. Alinaghi , M. Calov , L. E. Kristensen , et al., “Prevalence of Psoriatic Arthritis in Patients With Psoriasis: A Systematic Review and Meta‐Analysis of Observational and Clinical Studies,” Journal of the American Academy of Dermatology 80, no. 1 (2019): 251–265.e19, 10.1016/j.jaad.2018.06.027. 29928910

[2] L. C. Coates and P. S. Helliwell , “Psoriatic Arthritis: State of the Art Review,” Clinical Medicine 17, no. 1 (2017): 65–70, 10.7861/clinmedicine.17-1-65. PMC6297592 28148584

[3] M. Tan , J. Hu , H. Xiao , et al., “Concurrent Onset of Skin and Joint Symptoms Correlates With Higher Psoriatic Arthritis Disease Activity: A Single‐Center Retrospective Study,” Journal of the American Academy of Dermatology 89, no. 1 (2023): 173–175, 10.1016/j.jaad.2023.02.045. 36889647

[4] S. Gao and H. Song , “Differences Between Psoriatic Arthritis and Psoriasis in Multi‐Omics,” Archives of Dermatological Research 316, no. 6 (2024): 217, 10.1007/s00403-024-03018-9. 38787526

[5] L. Liu , X. C. Cai , X. Y. Sun , et al., “Global Prevalence of Metabolic Syndrome in Patients With Psoriasis in the past Two Decades: Current Evidence,” Journal of the European Academy of Dermatology and Venereology: JEADV 36, no. 11 (2022): 1969–1979, 10.1111/jdv.18296. 35666614

[6] R. A. Charlton , A. Green , G. Shaddick , et al., “Risk of Osteoarthritis in an Incident Cohort of People With Psoriatic Arthritis: A Population‐Based Cohort Study,” Journal of Rheumatology 48, no. 6 (2021): 841–846, 10.3899/jrheum.200564. 33191285

[7] L. Liu , N. X. Lin , Y. T. Yu , et al., “Epidemiology of Mental Health Comorbidity in Patients With Psoriasis: An Analysis of Trends From 1986 to 2019,” Psychiatry Research 321 (2023): 115078, 10.1016/j.psychres.2023.115078. 36724650

[8] R. Queiro , A. Lorenzo , E. Pardo , A. Brandy , P. Coto , and J. Ballina , “Prevalence and Type II Diabetes‐Associated Factors in Psoriatic Arthritis,” Clinical Rheumatology 37, no. 4 (2018): 1059–1064, 10.1007/s10067-018-4042-1. 29470738

[9] J. Ciaffi , G. Mele , L. Mancarella , et al., “Prevalence of Type 2 and Type 1 Diabetes in Psoriatic Arthritis: An Italian Study,” Journal of Clinical Rheumatology: practical reports on rheumatic & musculoskeletal diseases 28, no. 2 (2022): e324–e329, 10.1097/rhu.0000000000001706. 35192591

[10] American Diabetes Association Professional Practice Committee for D , “2. Diagnosis and Classification of Diabetes: Standards of Care in Diabetes—2026,” supplement, Diabetes Care 49, no. Supplement_1 (2025): S27–S49, 10.2337/dc26-S002. PMC11635041 39651986

[11] GBD 2021 Diabetes Collaborators , “Global, Regional, and National Burden of Diabetes From 1990 to 2021, With Projections of Prevalence to 2050: A Systematic Analysis for the Global Burden of Disease Study 2021,” Lancet 402, no. 10397 (2023): 203–234, https://pubmed.ncbi.nlm.nih.gov/37356446/. 10.1016/S0140-6736(23)01301-6 PMC10364581 37356446

[12] H. Furuya , C. T. Nguyen , T. Chan , et al., “IL‐23 Induces CLEC5A(+) IL‐17A(+) Neutrophils and Elicit Skin Inflammation Associated With Psoriatic Arthritis,” Journal of Autoimmunity 143 (2024): 103167, 10.1016/j.jaut.2024.103167. PMC10981569 38301504

[13] Y. Li , Z. Li , K. S. Nandakumar , and R. Holmdahl , “Human NCF1(90H) Variant Promotes IL‐23/IL‐17‐Dependent Mannan‐Induced Psoriasis and Psoriatic Arthritis,” Antioxidants 12, no. 7 (2023): 1348, 10.3390/antiox12071348. PMC10376330 37507888

[14] S. Rajendran , E. Quesada‐Masachs , S. Zilberman , et al., “IL‐17 is Expressed on Beta and Alpha Cells of Donors With Type 1 and Type 2 Diabetes,” Journal of Autoimmunity 123 (2021): 102708, 10.1016/j.jaut.2021.102708. PMC8386282 34358764

[15] A. Coomans de Brachène , M. I. Alvelos , F. Szymczak , et al., “Interferons are Key Cytokines Acting on Pancreatic Islets in Type 1 Diabetes,” Diabetologia 67, no. 5 (2024): 908–927, 10.1007/s00125-024-06106-7. 38409439

[16] I. Olejniczak‐Staruch , M. Ciążyńska , D. Sobolewska‐Sztychny , J. Narbutt , M. Skibińska , and A. Lesiak , “Alterations of the Skin and Gut Microbiome in Psoriasis and Psoriatic Arthritis,” International Journal of Molecular Sciences 22, no. 8 (2021): 3998, 10.3390/ijms22083998. PMC8069836 33924414

[17] Z. Ruan , T. Lu , Y. Chen , et al., “Association Between Psoriasis and Nonalcoholic Fatty Liver Disease Among Outpatient US Adults,” JAMA dermatology 158, no. 7 (2022): 745–753, 10.1001/jamadermatol.2022.1609. PMC9134040 35612851

[18] E. Gracey , L. Vereecke , D. McGovern , et al., “Revisiting the Gut‐Joint Axis: Links Between Gut Inflammation and Spondyloarthritis,” Nature Reviews Rheumatology 16, no. 8 (2020): 415–433, 10.1038/s41584-020-0454-9. 32661321

[19] Q. Wang , J. Wang , X. Sun , et al., “Evidence‐Based Dietary Recommendations for Patients With Psoriasis: A Systematic Review,” Clinical Nutrition 47 (2025): 68–82, 10.1016/j.clnu.2025.02.005. 39987781

[20] P. M. G. Acioly , M. Mazzillo , C. J. Machado , et al., “Metabolic Syndrome in Psoriasis and Psoriatic Arthritis in a Mixed Race Population: Comparison of Their Prevalences,” Psoriasis: Targets and Therapy 14 (2024): 123–130, 10.2147/ptt.s471707. PMC11533878 39498380

[21] K. Bonek , E. Kuca Warnawin , A. Kornatka , et al., “Circulating miRNA Correlates With Lipid Profile and Disease Activity in Psoriatic Arthritis, Rheumatoid Arthritis, and Ankylosing Spondylitis Patients,” Biomedicines 10, no. 4 (2022): 893, 10.3390/biomedicines10040893. PMC9024741 35453643

[22] Y. Gao , X. Yi , and Y. Ding , “Combined Transcriptomic Analysis Revealed AKR1B10 Played an Important Role in Psoriasis Through the Dysregulated Lipid Pathway and Overproliferation of Keratinocyte,” Biomed Research International 2017 (2017): 8717369, 10.1155/2017/8717369. PMC5674492 29204449

[23] F. Caso , L. Postiglione , B. Covelli , et al., “Pro‐Inflammatory Adipokine Profile in Psoriatic Arthritis: Results From a Cross‐Sectional Study Comparing PsA Subset With Evident Cutaneous Involvement and Subset Sine Psoriasis,” Clinical Rheumatology 38, no. 9 (2019): 2547–2552, 10.1007/s10067-019-04619-w. 31147798

[24] S. Słuczanowska‐Głabowska , M. Staniszewska , M. Marchlewicz , et al., “Adiponectin, Leptin and Resistin in Patients With Psoriasis,” Journal of Clinical Medicine 12, no. 2 (2023): 663, 10.3390/jcm12020663. PMC9860551 36675592

[25] M. Radić , A. Belančić , H. Đogaš , et al., “Cardiometabolic Risk in Psoriatic Arthritis: A Hidden Burden of Inflammation and Metabolic Dysregulation,” Metabolites 15, no. 3 (2025): 206, 10.3390/metabo15030206. PMC11943837 40137170

[26] S. M. Schlenker , S. I. Munhoz , A. R. Busanello , et al., “Resistin Serum Levels and its Association With Clinical Profile and Carotid Intima‐Media Thickness in Psoriasis: A Cross‐Sectional Study,” Anais Brasileiros de Dermatologia 98, no. 6 (2023): 799–805, 10.1016/j.abd.2022.10.011. PMC10589471 37355349

[27] H. M. Yang , J. Kim , B. K. Kim , et al., “Resistin Regulates Inflammation and Insulin Resistance in Humans via the Endocannabinoid System,” Research: Ideas for Today's Investors 7 (2024): 0326, 10.34133/research.0326. PMC11267475 39050819

[28] R. Belli , A. Dattolo , F. Sampogna , et al., “Leptin: A Gender and Obesity‐Related Marker Predictive of Metabolic Comorbidities and Therapeutic Response to anti‐IL‐23 Biologic Drugs in Psoriatic Patients,” Frontiers in Immunology 16 (2025): 1607312, 10.3389/fimmu.2025.1607312. PMC12307160 40740781

[29] N. Ahmed , J. A. Prior , Y. Chen , R. Hayward , C. D. Mallen , and S. L. Hider , “Prevalence of Cardiovascular‐Related Comorbidity in Ankylosing Spondylitis, Psoriatic Arthritis and Psoriasis in Primary Care: A Matched Retrospective Cohort Study,” Clinical Rheumatology 35, no. 12 (2016): 3069–3073, 10.1007/s10067-016-3362-2. 27485152

[30] M. T. Alrubaiaan , S. A. Alsulaiman , A. N. Altasan , A. Alqahtani , A. Alrashid , and O. L. Mohamed , “Prevalence of Cardiovascular Risk Factors in Saudi Patients With Psoriatic Arthritis: A Single‐Center Retrospective Cohort Study,” Cureus 15, no. 10 (2023): e46570, 10.7759/cureus.46570. PMC10625670 37933346

[31] F. Atzeni , L. La Corte , M. Cirillo , M. Giallanza , and J. Rodríguez‐Carrio , “Metabolic Syndrome and Its Components Have A Different Presentation and Impact on the Cardiovascular Risk Profile of Patients With Psoriatic and Rheumatoid Arthritis,” Annals of the Rheumatic Diseases 82 (2023): 1352, 10.3390/jcm12155031. PMC10420311 37568433

[32] S. Chandrashekara , P. Shenoy , U. Kumar , S. Pandya , and A. Ghosh , “Burden of Associated Comorbidities in Autoimmune Rheumatic Diseases in Indian Population: An Interim Report Based on the Indian Rheumatology Association Database,” Indian Journal of Rheumatology 17, no. 3 (2022): 227–233, 10.4103/injr.injr_125_21.

[33] R. Charlton , A. Green , G. Shaddick , et al., “Risk of Type 2 Diabetes and Cardiovascular Disease in an Incident Cohort of People With Psoriatic Arthritis: A Population‐Based Cohort Study,” Rheumatology 58, no. 1 (2019): 144–148, 10.1093/rheumatology/key286. 30202906

[34] J. Dreiher , T. Freud , and A. D. Cohen , “Psoriatic Arthritis and Diabetes: A Population‐Based Cross‐Sectional Study,” Dermatology Research and Practice 2013 (2013): 1–7, 10.1155/2013/580404. PMC3703365 23843781

[35] C. Edens and M. Antonelli , “The Prevalence of Comorbidities in Children and Young Adults With Psoriasis and Psoriatic Arthritis,” Journal of Psoriasis and Psoriatic Arthritis 4, no. 1 (2019): 22–27, 10.1177/2475530318807695.

[36] E. Edson‐Heredia , B. Zhu , C. Lefevre , et al., “Prevalence and Incidence Rates of Cardiovascular, Autoimmune, and Other Diseases in Patients With Psoriatic or Psoriatic Arthritis: A Retrospective Study Using Clinical Practice Research Datalink,” Journal of the European Academy of Dermatology and Venereology 29, no. 5 (2015): 955–963, 10.1111/jdv.12742. 25352213

[37] V. Furer , S. Shenhar‐Tsarfaty , S. Berliner , et al., “Prevalence of high‐sensitivity Cardiac Troponin T in real‐life Cohorts of Psoriatic Arthritis and General Population: A cross‐sectional Study,” Rheumatology International 40, no. 3 (2020): 437–444, 10.1007/s00296-019-04461-y. 31646356

[38] C. Han , D. W. Robinson Jr , M. V. Hackett , L. C. Paramore , K. H. Fraeman , and M. V. Bala , “Cardiovascular Disease and Risk Factors in Patients With Rheumatoid Arthritis, Psoriatic Arthritis, and Ankylosing Spondylitis,” Journal of Rheumatology 33, no. 11 (2006): 2167–2172, https://pubmed.ncbi.nlm.nih.gov/16981296/. 16981296

[39] N. Haque , R. J. Lories , and K. De Vlam , “Comorbidities Associated With Psoriatic Arthritis Compared With Non‐Psoriatic Spondyloarthritis: A Cross‐Sectional Study,” Journal of Rheumatology 43, no. 2 (2016): 376–382, 10.3899/jrheum.141359. 26669922

[40] R. Huang , Y. Zhuang , Y. Chang , L. F. Li , and H. J. Xu , “A Retrospective Analysis of Comorbidities in Patients With Psoriasis at a Single Centre,” Postępy Dermatologii i Alergologii 41, no. 2 (2024): 164–172, 10.5114/ada.2024.139123. PMC11110224 38784921

[41] K. Jafri , C. M. Bartels , D. Shin , J. M. Gelfand , and A. Ogdie , “Incidence and Management of Cardiovascular Risk Factors in Psoriatic Arthritis and Rheumatoid Arthritis: A Population‐Based Study,” Arthritis Care & Research 69, no. 1 (2017): 51–57, 10.1002/acr.23094. PMC5191972 27696731

[42] J. Kaine , X. Song , G. Kim , P. Hur , and J. B. Palmer , “Higher Incidence Rates of Comorbidities in Patients With Psoriatic Arthritis Compared With the General Population Using U.S. Administrative Claims Data,” Journal of Managed Care and Specialty Pharmacy 25, no. 1 (2019): 122–132, 10.18553/jmcp.2018.17421. PMC10397587 29694270

[43] A. J. Landgren , M. Dehlin , L. Jacobsson , U. Bergsten , and E. Klingberg , “Cardiovascular Risk Factors in Gout, Psoriatic Arthritis, Rheumatoid Arthritis and Ankylosing Spondylitis: A Cross‐Sectional Survey of Patients in Western Sweden,” RMD Open 7, no. 2 (2021): e001568, 10.1136/rmdopen-2021-001568. PMC8154995 34031261

[44] M. Mazzillo , P. G. Accioly , C. Camargo , et al., “Evaluation of Cardiovascular Risk in Patients With Psoriatic Disease in a Multiracial Population: A Cross‐Sectional Study,” Clinical and Experimental Dermatology 47, no. 9 (2022): 1666–1672, 10.1111/ced.15233. 35437808

[45] H. Radner , T. Lesperance , N. A. Accortt , and D. H. Solomon , “Incidence and Prevalence of Cardiovascular Risk Factors Among Patients With Rheumatoid Arthritis, Psoriasis, or Psoriatic Arthritis,” Arthritis Care & Research 69, no. 10 (2017): 1510–1518, 10.1002/acr.23171. 27998029

[46] M. Skornicki , P. Prince , R. Suruki , E. Lee , and A. Louder , “Clinical Burden of Concomitant Joint Disease in Psoriasis: A US‐Linked Claims and Electronic Health Records Database Analysis,” Advances in Therapy 38, no. 5 (2021): 2458–2471, 10.1007/s12325-021-01698-7. PMC8107168 33818686

[47] S. Urbancek , R. Sutka , Z. Kmecova , et al., “Screening of Patients With Psoriasis for Psoriatic Arthritis in the Slovak Republic,” Acta Medica Martiniana 16, no. 3 (2016): 32–42, 10.1515/acm-2016-0015.

[48] D. Vega‐Morales , A. Garza‐Alpirez , M. F. Martínez‐Guerra , and A. E. Montoya‐Montes , “Prevalence of Comorbidities and Use of Medication in Psoriatic Arthritis Patients: Findings From a Mexican Cohort,” Revista Colombiana de Reumatología (2024), https://www.sciencedirect.com/science/article/abs/pii/S0121812324000641.

[49] D. Zisman , R. I. Ashkenazi , H. Bitterman , et al., “Endocrine Co‐Morbidities in Patients With Psoriatic Arthritis: A Population‐Based Study,” Arthritis & Rheumatology 66 (2014): S682, https://acrabstracts.org/abstract/endocrine‐co‐morbidities‐in‐patients‐with‐psoriatic‐arthritis‐a‐population‐based‐study/.

[50] R. A. L. Johani , A. Polachek , S. Li , V. Chandran , and D. D. Gladman , “Characteristic and Outcomes of Psoriatic Arthritis Patients With Hyperuricemia, Whether we Should Treat or not?,” Arthritis & Rheumatology 68 (2016): 3695–3697, https://pubmed.ncbi.nlm.nih.gov/29196385/.

[51] A. Kibari , A. D. Cohen , T. Gazitt , et al., “Cardiac and Cardiovascular Morbidities in Patients With Psoriatic Arthritis: A Population‐Based Case Control Study,” Clinical Rheumatology 38, no. 8 (2019): 2069–2075, 10.1007/s10067-019-04528-y. 30937638

[52] L. Puig , R. Strohal , M. E. Husni , et al., “Cardiometabolic Profile, Clinical Features, Quality of Life and Treatment Outcomes in Patients With Moderate‐to‐Severe Psoriasis and Psoriatic Arthritis,” Journal of Dermatological Treatment 26, no. 1 (2015): 7–15, 10.3109/09546634.2013.860209. 24283931

[53] S. Chandrashekara , P. Shenoy , U. Kumar , et al., “Increase in Comorbidities With Age Among Patients With Psoriatic Arthritis: A Multicenter Observational Study,” Rheumatology International 45, no. 1 (2025): 23, 10.1007/s00296-024-05760-9. 39786433

[54] J. Li , J. Xiao , X. Xie , et al., “Individual Joints Involvement Pattern in Psoriatic Arthritis: A Cross‐Sectional Study in China,” Journal of Dermatology 51, no. 12 (2024): 1607–1614, 10.1111/1346-8138.17369. 38995193

[55] S. Koppikar , B. Kuriya , J. A. Udell , et al., “Risk of Heart Failure in Patients With Immune‐Mediated Inflammatory Diseases: A Population‐Based Study,” Journal of Rheumatology (2025), https://pubmed.ncbi.nlm.nih.gov/40041997/. 10.3899/jrheum.2024-0866 40041997

[56] S. Alonso , P. Tejón , C. Sarasqueta , P. Coto , M. Alperi , and R. Queiro , “Age at Disease Onset may Help to Further Characterize the Disease Phenotype in Psoriatic Arthritis,” Joint Bone Spine 83, no. 5 (2016): 533–537, 10.1016/j.jbspin.2015.09.004. 26750761

[57] Y. Degboé , R. Koch , L. Zabraniecki , et al., “Increased Cardiovascular Risk in Psoriatic Arthritis: Results From a Case‐Control Monocentric Study,” Frontiers of Medicine 9 (2022): 785719, 10.3389/fmed.2022.785719. PMC9160333 35665348

[58] L. Eder , A. Thavaneswaran , V. Chandran , R. Cook , and D. D. Gladman , “Increased Burden of Inflammation Over Time is Associated With the Extent of Atherosclerotic Plaques in Patients With Psoriatic Arthritis,” Annals of the Rheumatic Diseases 74, no. 10 (2015): 1830–1835, 10.1136/annrheumdis-2014-205267. 24827532

[59] L. Eder , Y. Wu , V. Chandran , R. Cook , and D. D. Gladman , “Incidence and Predictors for Cardiovascular Events in Patients With Psoriatic Arthritis,” Annals of the Rheumatic Diseases 75, no. 9 (2016): 1680–1686, 10.1136/annrheumdis-2015-207980. 26493817

[60] M. H. Favarato , P. Mease , C. R. Gonçalves , C. Gonçalves Saad , P. D. Sampaio‐Barros , and C. Goldenstein‐Schainberg , “Hypertension and Diabetes Significantly Enhance the Risk of Cardiovascular Disease in Patients With Psoriatic Arthritis,” Clinical & Experimental Rheumatology 32, no. 2 (2014): 182–187, https://pubmed.ncbi.nlm.nih.gov/24480317/. 24480317

[61] S. F. Goh , S. B. Wong , S. Robinson , and M. M. Tang , “Clinical Profile, Treatment and Quality of Life of Patients With Psoriatic Arthritis in Malaysia: A Population‐Based Cross‐Sectional Study,” Experimental Dermatology 33, no. 3 (2024): e15060, 10.1111/exd.15060. 38532576

[62] A. Ishchenko , S. Pazmino , B. Neerinckx , R. Lories , and K. de Vlam , “Comorbidities in Early Psoriatic Arthritis: Data From the Metabolic Disturbances in Psoriatic Arthritis Cohort Study,” Arthritis Care & Research 76, no. 2 (2024): 231–240, 10.1002/acr.25230. 37667975

[63] K. Kamiya and M. Ohtsuki , “Epidemiological Survey of Patients With Psoriatic Arthritis in the Japanese Society for Psoriasis Research From 2017 to 2020,” Journal of Dermatology 50, no. 1 (2023): 12–25, 10.1111/1346-8138.16603. PMC10092149 36261862

[64] M. Labitigan , A. Bahče‐Altuntas , J. M. Kremer , et al., “Higher Rates and Clustering of Abnormal Lipids, Obesity, and Diabetes Mellitus in Psoriatic Arthritis Compared With Rheumatoid Arthritis,” Arthritis Care & Research 66, no. 4 (2014): 600–607, 10.1002/acr.22185. PMC3969762 24115739

[65] K. Nas , M. Karkucak , B. Durmus , et al., “Comorbidities in Patients With Psoriatic Arthritis: A Comparison With Rheumatoid Arthritis and Psoriasis,” International journal of rheumatic diseases 18, no. 8 (2015): 873–879, 10.1111/1756-185x.12580. 26173043

[66] P. S. Ong , L. K. Tan , H. Mat , et al., “Factors Influencing Liver Abnormalities in Psoriatic Arthritis Patients: A Comprehensive Study,” Mediterranean Journal of Rheumatology 35, no. 2 (2024): 234–240, 10.31138/mjr.050723.fla. PMC11350418 39211017

[67] E. Papadavid , P. Katsimbri , I. Kapniari , et al., “Prevalence of Psoriatic Arthritis and Its Correlates Among Patients With Psoriasis in Greece: Results From a Large Retrospective Study,” Journal of the European Academy of Dermatology and Venereology 30, no. 10 (2016): 1749–1752, 10.1111/jdv.13700. 27508394

[68] L. Pina Vegas , E. Sbidian , L. Penso , and P. Claudepierre , “Epidemiologic Study of Patients With Psoriatic Arthritis in a Real‐World Analysis: A Cohort Study of the French Health Insurance Database,” Rheumatology 60, no. 3 (2021): 1243–1251, 10.1093/rheumatology/keaa448. 32911531

[69] A. Polat Ekinci , G. Pehlivan , and M. O. Gokalp , “Surveillance of Psoriatic Patients on Biologic Treatment During the COVID‐19 Pandemic: A Single‐Center Experience,” Dermatologic Therapy 34, no. 1 (2021): e14700, 10.1111/dth.14700. 33369063

[70] C. T. Ritchlin , M. Stahle , Y. Poulin , et al., “Serious Infections in Patients With Self‐Reported Psoriatic Arthritis From the Psoriasis Longitudinal Assessment and Registry (PSOLAR) Treated With Biologics,” BMC Rheumatology 3, no. 1 (2019): 52, 10.1186/s41927-019-0094-3. PMC6882230 31799498

[71] Y. Ross , S. Jaleel , and M. Magrey , “Racial Disparities in Comorbidities of Patients With Psoriatic Arthritis,” Rheumatology International 43, no. 8 (2023): 1525–1529, 10.1007/s00296-023-05322-5. 37017711

[72] S. Scriffignano , R. Lories , A. Nzeusseu Toukap , et al., “Cardiovascular Comorbidities in Psoriatic Arthritis: Epidemiology and Risk Factors in Two Different European Populations,” Clinical & Experimental Rheumatology 41, no. 9 (2023): 1815–1822, https://pubmed.ncbi.nlm.nih.gov/36826796/. 10.55563/clinexprheumatol/aovika 36826796

[73] K. Shah , M. Paris , L. Mellars , A. Changolkar , and P. J. Mease , “Real‐World Burden of Comorbidities in US Patients With Psoriatic Arthritis,” RMD Open 3, no. 2 (2017): e000588, 10.1136/rmdopen-2017-000588. PMC5761305 29435363

[74] P. Sinnathurai , R. Buchbinder , C. Hill , M. Lassere , and L. March , “Comorbidity in Psoriatic Arthritis and Rheumatoid Arthritis,” Internal Medicine Journal 48, no. 11 (2018): 1360–1368, 10.1111/imj.14046. 30047189

[75] Z. Song , X. Deng , W. Xie , B. Li , and Z. Zhang , “Clinical Characteristics of Psoriatic Arthritis in Chinese Patients: A Cross‐Sectional Study,” Rheumatology and Therapy 8, no. 4 (2021): 1845–1857, 10.1007/s40744-021-00384-y. PMC8572294 34665456

[76] C. S. Souza , C. C. S. de Castro , F. R. O. Carneiro , et al., “Metabolic Syndrome and Psoriatic Arthritis Among Patients With Psoriasis Vulgaris: Quality of Life and Prevalence,” Journal of Dermatology 46, no. 1 (2019): 3–10, 10.1111/1346-8138.14706. PMC6587528 30474868

[77] L. S. Tam , B. Tomlinson , T. T. Chu , et al., “Cardiovascular Risk Profile of Patients With Psoriatic Arthritis Compared to controls—The Role of Inflammation,” Rheumatology 47, no. 5 (2008): 718–723, 10.1093/rheumatology/ken090. 18400833

[78] N. F. Velez , E. X. Wei‐Passanese , M. E. Husni , E. A. Mody , and A. A. Qureshi , “Management of Psoriasis and Psoriatic Arthritis in a Combined Dermatology and Rheumatology Clinic,” Archives of Dermatological Research 304, no. 1 (2012): 7–13, 10.1007/s00403-011-1172-6. 21904925

[79] D. Zisman , L. Eder , M. Elias , et al., “Clinical and Demographic Characteristics of Patients With Psoriatic Arthritis in Northern Israel,” Rheumatology International 32, no. 3 (2012): 595–600, 10.1007/s00296-010-1673-1. 21120489

[80] L. Eder , P. Harvey , V. Chandran , et al., “Gaps in Diagnosis and Treatment of Cardiovascular Risk Factors in Patients With Psoriatic Disease: An International Multicenter Study,” Journal of Rheumatology 45, no. 3 (2018): 378–384, 10.3899/jrheum.170379. PMC5845845 29419462

[81] H. G. Tekin , J. J. Wu , R. Burge , J. Birt , and A. Egeberg , “Burden and Disease Characteristics of Patients With Psoriatic Arthritis: A Population‐Based Cross‐Sectional Study,” Journal of Rheumatology 46, no. 7 (2019): 716–720, 10.3899/jrheum.180670. 30824662

[82] G. Alp , M. Kara , and H. Cinakli , “Potential Difficult‐To‐Treat Psoriatic Arthritis Real‐World Prevalence And Contributing Factors,” Annals of the Rheumatic Diseases 83 (2024): 1481, https://pubmed.ncbi.nlm.nih.gov/39152760/. 10.55563/clinexprheumatol/pqpzef 39152760

[83] D. Freites‐Nuñez , L. Leon , E. Toledano , et al., “Switching Related to Inefficacy in Biologics and Targeted Synthetic Therapies for Psoriatic Arthritis: A Comparative Real‐Life Study,” Therapeutic Advances in Musculoskeletal Disease 16 (2024): 1759720X241273083, 10.1177/1759720x241273083. PMC11366104 39219744

[84] F. Kharouf , S. Gao , S. Al‐Matar , R. J. Cook , V. Chandran , and D. D. Gladman , “Chronic Kidney Disease in Patients With Psoriatic Arthritis: A Cohort Study,” RMD Open 10, no. 4 (2024): e004636, 10.1136/rmdopen-2024-004636. PMC11552569 39510763

[85] Z. Guła , K. Łosińska , P. Kuszmiersz , et al., “A Comparison of Comorbidities and Their Risk Factors Prevalence Across Rheumatoid Arthritis, Psoriatic Arthritis and Axial Spondyloarthritis With Focus on Cardiovascular Diseases: Data From a Single Center Real‐World Cohort,” Rheumatology International 44, no. 12 (2024): 2817–2828, 10.1007/s00296-024-05740-z. PMC11618134 39527279

[86] W. Pei , L. Xu , H. Zhong , et al., “Clinical Features of Inflammatory Arthritis in Daily Practice‐china's Perspective,” Clinical Rheumatology 44, no. 3 (2025): 969–978, 10.1007/s10067-024-07262-2. 39853560

[87] R. Queiro , A. Lorenzo , P. Tejon , E. Pardo , P. Coto , and J. Ballina , “Polyarticular Evolution and Late‐Onset Psoriasis may be Associated With Cardiovascular Disease in Psoriatic Arthritis,” International journal of rheumatic diseases 22, no. 2 (2019): 269–274, 10.1111/1756-185x.13421. 30398011

[88] M. Khraishi , D. MacDonald , E. Rampakakis , J. Vaillancourt , and J. S. Sampalis , “Prevalence of Patient‐Reported Comorbidities in Early and Established Psoriatic Arthritis Cohorts,” Clinical Rheumatology 30, no. 7 (2011): 877–885, 10.1007/s10067-011-1692-7. 21287359

[89] M. Khraishi , R. Aslanov , E. Rampakakis , C. Pollock , and J. S. Sampalis , “Prevalence of Cardiovascular Risk Factors in Patients With Psoriatic Arthritis,” Clinical Rheumatology 33, no. 10 (2014): 1495–1500, 10.1007/s10067-014-2743-7. 25034080

[90] N. Fathallah , R. Slim , S. Larif , H. Hmouda , and C. Ben Salem , “Drug‐Induced Hyperglycaemia and Diabetes,” Drug Safety 38, no. 12 (2015): 1153–1168, 10.1007/s40264-015-0339-z. 26370106

[91] J. L. Hwang and R. E. Weiss , “Steroid‐Induced Diabetes: A Clinical and Molecular Approach to Understanding and Treatment,” Diabetes/metabolism research and reviews 30, no. 2 (2014): 96–102, 10.1002/dmrr.2486. PMC4112077 24123849

[92] P. Coto‐Segura , N. Eiris‐Salvado , L. González‐Lara , et al., “Psoriasis, Psoriatic Arthritis and Type 2 Diabetes Mellitus: A Systematic Review and Meta‐Analysis,” British Journal of Dermatology 169, no. 4 (2013): 783–793, 10.1111/bjd.12473. 23772556

[93] Z. Yuan and Y. Guo , “Risk of Incident Type 2 Diabetes in Patients With Psoriatic Arthritis: A Systematic Review and Meta‐Analysis of Cohort Studies,” International journal of rheumatic diseases 25, no. 9 (2022): 1029–1037, 10.1111/1756-185x.14375. 35766111

[94] J. J. Noubiap , J. R. Nansseu , E. Lontchi‐Yimagou , et al., “Geographic Distribution of Metabolic Syndrome and Its Components in the General Adult Population: A Meta‐Analysis of Global Data From 28 Million Individuals,” Diabetes Research and Clinical Practice 188 (2022): 109924, 10.1016/j.diabres.2022.109924. 35584716

[95] L. A. Parra‐Gómez , J. P. Puerta Rojas , A. J. Vásquez , et al., “Prevalence of Metabolic Syndrome in Latin America: A Systematic Review and Meta‐Analysis of Observational Studies,” Diabetes & Metabolic Syndrome 19, no. 7 (2025): 103282, 10.1016/j.dsx.2025.103282. 40865354

[96] Y. Nie , H. Zhou , J. Wang , and H. Kan , “Association Between Systemic Immune‐Inflammation Index and Diabetes: A Population‐Based Study From the NHANES,” Frontiers in Endocrinology 14 (2023): 1245199, 10.3389/fendo.2023.1245199. PMC10644783 38027115

[97] K. Huang , Y. Liang , Y. Ma , J. Wu , H. Luo , and B. Yi , “The Variation and Correlation of Serum Adiponectin, Nesfatin‐1, IL‐6, and TNF‐α Levels in Prediabetes,” Frontiers in Endocrinology 13 (2022): 774272, 10.3389/fendo.2022.774272. PMC8928772 35311231

[98] D. Qu , J. Liu , C. W. Lau , and Y. Huang , “IL‐6 in Diabetes and Cardiovascular Complications,” British Journal of Pharmacology 171, no. 15 (2014): 3595–3603, 10.1111/bph.12713. PMC4128059 24697653

[99] Y. Li , X. Chen , Y. Chen , et al., “Berberine Improves TNF‐α‐Induced Hepatic Insulin Resistance by Targeting MEKK1/MEK Pathway,” Inflammation 45, no. 5 (2022): 2016–2026, 10.1007/s10753-022-01671-8. 35460012

[100] M. Tesi , M. Bugliani , G. Ferri , et al., “Pro‐Inflammatory Cytokines Induce Insulin and Glucagon Double Positive Human Islet Cells That Are Resistant to Apoptosis,” Biomolecules 11, no. 2 (2021): 320, 10.3390/biom11020320. PMC7923272 33669901

[101] F. Ding , X. Luo , Y. Tu , et al., “Alpk1 Sensitizes Pancreatic Beta Cells to Cytokine‐Induced Apoptosis via Upregulating TNF‐α Signaling Pathway,” Frontiers in Immunology 12 (2021): 705751, 10.3389/fimmu.2021.705751. PMC8490819 34621265

[102] A. Kumar , K. Sundaram , J. Mu , et al., “High‐Fat Diet‐Induced Upregulation of Exosomal Phosphatidylcholine Contributes to Insulin Resistance,” Nature Communications 12, no. 1 (2021): 213, 10.1038/s41467-020-20500-w. PMC7801461 33431899

