# Supplementary material for: Epidemiology of Diabetes Mellitus in Patients With Psoriatic Arthritis: Current Evidence
Source: Diabetes Metab Res Rev. 2026 Oct 27;42(7):e70239. doi: 10.1002/dmrr.70239 (PMC13711012; doi:10.1002/dmrr.70239)
Supplement: Supplementary file 1 [file DMRR-42-e70239-s001.docx]

**Table S3. Characteristics of the included studies without treatment disclosure**

| **Author- publication year** | **Study year** | **Study type** | **Region** | **F/M** | **Age[Mean±SD]** | **DM type** | **PsA(n)** | **Comorbidities (n)** |
| --- | --- | --- | --- | --- | --- | --- | --- | --- |
| Ahmed, Nadeem et al.2016 | 1999-2009 | Cohort study | Britain | 51/55 | 59.5±13.8 | DM | 106 | 12 |
| Alrubaiaan, Mishari T et al.2023 | 2015-2023 | Cohort study | Saudi Arabia | 37/89 | 42.4±13.2 | DM | 126 | 38 |
| Atzeni, Fabiola et al.2023 | 2021 | Cross-sectional study | Italy | 24/54 | 45.23 ±16.99 | DM | 78 | 26 |
| Chandrashekara, Srikantiah et al.2022 | 2021 | Cross-sectional study | India | 61/44 | 48.65 ±13.35 | DM | 105 | 17 |
| Charlton, Rachel et al.2019 | 1998-2014 | Cohort study | Britain | 3327/3456 | - | T2DM | 6783 | 414 |
| Dreiher, Jacob et al.2013 | - | Cross-sectional study | Israel | 249/300 | 53.8±14.1 | DM | 549 | 84 |
| Dreiher, Jacob et al.2013 | - | Cross-sectional study | Israel | 249 | 53.8±14.1 | DM | 249 | 28 |
| Dreiher, Jacob et al.2013 | - | Cross-sectional study | Israel | 300 | 53.8±14.1 | DM | 300 | 56 |
| Edens, Cuoghi et al.2018 (1) | 1999-2018 | Cross-sectional study | America | 170/350 | - | T1DM | 520 | 10(Actual number between 10 and 20) |
| Edens, Cuoghi et al.2018 (2) | 1999-2018 | Cross-sectional study | America | 170/350 | - | T2DM | 520 | 30 |
| Edson-Heredia, E et al.2015 | 2006-2010 | Cohort study | Britain | 955/997 | 48.6±13.9 | T2DM | 1952 | 98 |
| Furer, Victoria et al. 2020 | 2012-2018 | Cross-sectional study | Israel | 57/60 | 51.0±12.9 | DM | 116 | 28 |
| Han, Chenglong et al.2006 | 2001-2002 | Cross-sectional study | America | 1505/1561 | 49.7±11.2 | T2DM | 3066 | 346 |
| Haque, Naba et al.2016 | 2000-2012 | Cross-sectional study | Belgium | 158/108 | 58.8±12.03 | DM | 262 | 17 |
| Huang, Rui et al.2024 | 2013-2023 | Cross-sectional study | China | 52/43 | - | T2DM | 95 | 28 |
| Jafri, Kashif et al.2017 | 1994-2014 | Cohort study | Britain | 6326/6222 | 50.07±14.28 | DM | 12548 | 973 |
| Kaine, Jeffrey et al.2019 | 2008-2015 | Cohort study | America | 6644/8254 | 53.4±12.4 | DM | 14898 | 2365 |
| Landgren, Anton Jonatan et al.2021 | 2015-2017 | Cross-sectional study | Sweden | 328/371 | 56.6±13.2 | DM | 699 | 72 |
| Mazzillo, Mara et al.2022 | 2019-2020 | Cross-sectional study | Brazil | 20/43 | 58±10.4 | DM | 63 | 26 |
| Radner, Helga et al.2017 (1) | 2002-2014 | Cohort study | America | 9372/9552 | 50.1±12.5 | DM | 18924 | 1476 |
| Skornicki, Michelle et al.2021 | 2007-2018 | Cross-sectional study | America | 1011/1300 | 46.3 ± 11.2 | DM | 2311 | 272 |
| Urbancek, Slavo et al.2016 | 2014-2015 | Cross-sectional study | Slovak Republic | 420/411 | 40-59 | DM | 177 | 34 |
| Vega-Morales, David et al. 2024 | 2017-2021 | Cross-sectional study | Mexico | 28/38 | 47.8±11.5 | DM | 66 | 23 |
| Haddad, Amir et al.2017 | 2002-2014 | Case-control study | Israel | 1474/1687 | 58.36±15.42 | DM | 3161 | 881 |
| AlJohani, RoaA et al.2018 (1) | 2006 | Case-control study | Canada | 192/126 | 51.4±13.3 | DM | 318 | 25 |
| AlJohani, RoaA et al.2018 (2) | 2006 | Case-control study | Canada | 192/126 | 53.4±13.7 | DM | 318 | 48 |
| Kibari, Adi et al.2019 | 2000-2013 | Case-control study | Israel | 1474/1987 | 58.36±15.42 | T2DM | 3161 | 881 |
| Puig, Luis et al.2013 |  | Cross-sectional study | America | 59/25 | 46.4±11.6 | DM | 84 | 18 |
| Chandrashekara, S et al.2025 | 2020 | Cross-sectional study | India | 274/259 | - | DM | 533 | 81 |
| Li, Jiashuai et al.2024 | 2019-2023 | Cross-sectional study | China | 156/108 | 46.7±13.1 | T2DM | 264 | 20 |
| Koppikar, Sahil et al.2025 | 2011-2019 | Cohort study | Canada | 3810/3840 | 55.0±13.3 | DM | 7650 | 1136 |

**Abbreviation:** PsA, Psoriatic Arthritis; F/M, Female/Male; DM, Diabetes Mellitus; T1DM, Type 1 Diabetes Mellitus; T2DM, Type 2 Diabetes Mellitus
