# Supplementary material for: Epidemiology of Diabetes Mellitus in Patients With Psoriatic Arthritis: Current Evidence
Source: Diabetes Metab Res Rev. 2026 Oct 27;42(7):e70239. doi: 10.1002/dmrr.70239 (PMC13711012; doi:10.1002/dmrr.70239)
Supplement: Supplementary file 3 [file DMRR-42-e70239-s003.docx]

| **Table S5. Risk of bias of the treatment-disclosed cross-sectional studies assessed using the Agency for Healthcare Research and Quality tool** | | | | | | | | | | | | |
| --- | --- | --- | --- | --- | --- | --- | --- | --- | --- | --- | --- | --- |
| ***Studies*** | ***Q1*** | ***Q2*** | ***Q3*** | ***Q4*** | ***Q5*** | ***Q6*** | ***Q7*** | ***Q8*** | ***Q9*** | ***Q10*** | ***Q11*** | ***Scores*** |
| Ciaffi, Jacopo et al.2022 | Y | Y | Y | Y | N | Y | U | Y | Y | Y | U | 8 |
| Goh, Shiau Fui et al.2024 | Y | Y | Y | Y | N | Y | Y | Y | N | U | U | 7 |
| Kamiya, Koji et al.2023 | Y | N | Y | U | N | N | U | Y | U | Y | U | 4 |
| Labitigan, Monalyn et al.2014 | Y | Y | Y | U | N | Y | U | Y | U | Y | U | 6 |
| Papadavid, E et al.2016 | Y | Y | Y | U | N | Y | U | Y | U | U | U | 5 |
| Polat Ekinci, Algün et al.2021 | Y | Y | Y | N | Y | N | N | N | U | N | U | 4 |
| Queiro, Rubén et al.2018 | Y | Y | Y | Y | N | Y | Y | Y | N | Y | Y | 9 |
| Queiro, Rubén et al.2019 | Y | Y | Y | Y | N | Y | Y | Y | N | Y | Y | 9 |
| Ross, Yael et al.2023 | Y | Y | Y | U | N | Y | N | Y | U | N | U | 5 |
| Scriffignano, Silvia et al.2023 | Y | Y | Y | N | N | Y | Y | Y | N | N | U | 6 |
| Song, Zhibo et al.2021 | Y | Y | Y | U | N | Y | Y | Y | N | N | U | 6 |
| Souza, Cacilda S et al. | Y | Y | Y | U | N | Y | Y | Y | U | Y | Y | 8 |
| Tam, L-S et al.2008 | Y | Y | N | Y | N | Y | U | U | U | Y | U | 5 |
| Velez, Nicole F et al.2012 | Y | Y | Y | Y | N | U | Y | Y | U | N | U | 6 |
| Zisman, Devy et al.2012 | Y | Y | Y | U | N | Y | N | Y | N | U | N | 5 |
| Eder, Lihi et al.2018 | Y | Y | Y | U | N | U | Y | Y | Y | U | N | 6 |
| Tekin, Hasan Gokcer et al.2019 | Y | Y | Y | Y | N | U | Y | U | U | Y | N | 6 |
| Khraishi, Majed et al.2014 | Y | Y | Y | U | N | Y | Y | Y | U | U | U | 6 |
| Khraishi, Majed et al.2011 | Y | Y | Y | N | N | Y | Y | Y | Y | U | U | 7 |
| Guła, Zofia et al.2024 | Y | Y | Y | N | U | Y | Y | U | Y | U | U | 6 |
| Pei, Wenwen et al.2025 | Y | Y | Y | U | N | N | Y | N | Y | Y | N | 6 |
| Questions (Q):  Q1 Define the source of information (survey, record review)  Q2 List inclusion and exclusion criteria for exposed and unexposed subjects (cases and controls) or refer to previous publications  Q3 Indicate time period used for identifying patients  Q4 Indicate whether or not subjects were consecutive if not population-based  Q5 Indicate if evaluators of subjective components of study were masked to other aspects of the status of the participants  Q6 Describe any assessments undertaken for quality assurance purposes (e.g., test/retest of primary outcome measurements)  Q7 Explain any patient exclusions from analysis  Q8 Describe how confounding was assessed and/or controlled  Q9 If applicable, explain how missing data were handled in the analysis  Q10 Summarize patient response rates and completeness of data collection  Q11 Clarify what follow-up, if any, was expected and the percentage of patients for which incomplete data or follow-up was obtained | | | | | | | | | | | | |
| Y, Yes; N, No; U, Unclear | | | | | | | | | | | | |
