# Supplementary material for: Epidemiology of Diabetes Mellitus in Patients With Psoriatic Arthritis: Current Evidence
Source: Diabetes Metab Res Rev. 2026 Oct 27;42(7):e70239. doi: 10.1002/dmrr.70239 (PMC13711012; doi:10.1002/dmrr.70239)
Supplement: Supplementary file 4 [file DMRR-42-e70239-s004.docx]

**MOOSE (Meta-analyses Of Observational Studies in Epidemiology) Checklist**

A reporting checklist for Authors, Editors, and Reviewers of Meta-analyses of Observational Studies. You must report the page number in your manuscript where you consider each of the items listed in this checklist. If you have not included this information, either revise your manuscript accordingly before submitting or note N/A.

| **Reporting Criteria** | **Reported (Yes/No)** | | | **Reported on Page No.** | | |
| --- | --- | --- | --- | --- | --- | --- |
| **Reporting of Background** |  | | |  | | |
| Problem definition |  | Yes |  |  | 4 |  |
| Hypothesis statement |  | No |  |  |  |  |
| Description of Study Outcome(s) |  | Yes |  |  | 4-5 |  |
| Type of exposure or intervention used |  | Yes |  |  | 4-6 |  |
| Type of study design used |  | Yes |  |  | 4-5 |  |
| Study population |  | Yes |  |  | 4-6 |  |
| **Reporting of Search Strategy** |  | | |  | | |
| Qualifications of searchers (eg, librarians  and investigators) | No | | |  | | |
| Search strategy, including time period  included in the synthesis and keywords | Yes | | | 4 | | |
| Effort to include all available studies,  including contact with authors | No | | |  | | |
|  |  |  |  |  |  |  |
| Databases and registries searched |  | Yes |  |  | 4 |  |
| Search software used, name and version, including special features used  (eg, explosion) | No | | |  | | |
| Use of hand searching (eg, reference  lists of obtained articles) | No | | |  | | |
| List of citations located and those  excluded, including justification | Yes | | | 4-5 | | |
| Method for addressing articles  published in languages other than English | Yes | | | 4-5 | | |
| Method of handling abstracts and  unpublished studies | Yes | | | 4-5 | | |
| Description of any contact with authors |  | No |  |  |  |  |
| **Reporting of Methods** |  | | |  | | |
| Description of relevance or appropriateness of studies assembled for  assessing the hypothesis to be tested | No | | |  | | |
| Rationale for the selection and coding of data (eg, sound clinical principles or  convenience) | No | | |  | | |
| Documentation of how data were classified and coded (eg, multiple raters,  blinding, and interrater reliability) | Yes | | | 5 | | |
| Assessment of confounding (eg, comparability of cases and controls in  studies where appropriate | No | | |  | | |

| **Reporting Criteria** | **Reported (Yes/No)** | | | **Reported on Page No.** | | |
| --- | --- | --- | --- | --- | --- | --- |
| Assessment of study quality, including blinding of quality assessors; stratification or regression on possible  predictors of study results | Yes | | | 5 | | |
| Assessment of heterogeneity |  | Yes |  |  | 5-6 |  |
| Description of statistical methods (eg, complete description of fixed or random effects models, justification of whether the chosen models account for predictors of study results, dose-response models, or cumulative meta-analysis) in sufficient  detail to be replicated | Yes | | | 5-6 | | |
| Provision of appropriate tables and  graphics | Yes | | | 22-26 | | |
| **Reporting of Results** |  | | |  | | |
| Table giving descriptive information for  each study included | Yes | | | TableS3-S4 | | |
| Results of sensitivity testing (eg,  subgroup analysis) | Yes | | | 7-11 | | |
| Indication of statistical uncertainty of  findings | Yes | | | 7-11 | | |
| **Reporting of Discussion** |  | | |  | | |
| Quantitative assessment of bias (eg,  publication bias) | Yes | | | 9, 11 | | |
| Justification for exclusion (eg, exclusion  of non–English-language citations) | Yes | | | 4–5 | | |
| Assessment of quality of included studies |  | Yes |  |  | 6-7 |  |
| **Reporting of Conclusions** |  | | |  | | |
| Consideration of alternative explanations  for observed results | No | | |  | | |
| Generalization of the conclusions (ie, appropriate for the data presented and  within the domain of the literature review) | Yes | | | 11-14 | | |
| Guidelines for future research |  | Yes |  |  | 13-14 |  |
| Disclosure of funding source |  | Yes |  |  | 1 |  |

**Once you have completed this checklist, please save a copy and upload it as part of your submission. DO NOT include this checklist as part of the main manuscript document. It must be uploaded as a separate file.**
