# Supplementary material for: Epidemiology of Diabetes Mellitus in Patients With Psoriatic Arthritis: Current Evidence
Source: Diabetes Metab Res Rev. 2026 Oct 27;42(7):e70239. doi: 10.1002/dmrr.70239 (PMC13711012; doi:10.1002/dmrr.70239)
Supplement: Supplementary file 5 [file DMRR-42-e70239-s005.docx]

| **Table S6. Quality assessment of the treatment-disclosed cohort and case-control studies using the Newcastle-Ottawa Scale** | | | | | | | | | |
| --- | --- | --- | --- | --- | --- | --- | --- | --- | --- |
| **1. Cohort studies** | | | | | | | | | |
| ***Studies*** | ***Selection*** | | | | ***Comparability*** | ***Outcome*** | | | ***Total score*** |
|  | ***Representativeness of the exposed cohort*** | ***Selection of the non-exposed cohort*** | ***Ascertainment of exposure*** | ***Demonstration that the outcome of interest was not present at the start of study*** | ***Comparability of cohorts on the basis of the design or analysis*** | ***Assessment of outcome*** | ***Follow-up long enough for outcomes to occur*** | ***Adequacy of follow-up of cohorts*** |  |
| Alonso, Sara et al.2016 | * | * | * | * |  | * | * | * | 7 |
| Eder, Lihi et al.2015 | * | * | * | * | * | * | * |  | 7 |
| Eder, Lihi et al.2016 | * | * | * | * | * | * | * | * | 8 |
| Favarato, Maria Helena et al.2014 | * | * | * | * | * |  |  |  | 5 |
| Ishchenko, Alla et al.2024 | * |  | * | * | * | * | * | * | 7 |
| Ong, Ping Seung et al.2023 | * | * | * | * | * | * | * | * | 8 |
| Pina Vegas, Laura et al.2021 | * | * | * | * | * | * | * | * | 8 |
| Ritchlin, Christopher T et al.2019 | * |  | * | * |  | * | * | * | 6 |
| Sinnathurai, Premarani et al.2018 | * | * | * | * | * | * |  |  | 6 |
| Alp, Gulay et al.2025 | * | * | * | * | * | * | * | * | 8 |
| Freites-Nunez, Dalifer et al.2024 | * | * | * | * | * |  | * |  | 6 |
| Kharouf, Fadi et al.2024 | * | * | * | * | * | * | * | * | 8 |
| **Case-control studies** | | | | | | | | | |
| ***Studies*** | ***Selection*** | | | | ***Comparability*** | ***Exposure*** | | | ***Total score*** |
|  | ***Is the case definition adequate*** | ***Representativeness of the cases*** | ***Selection of controls*** | ***Definition of controls*** | ***Comparability of cases and controls on the basis of the design or analysis*** | ***Ascertainment of exposure*** | ***Same method of ascertainment for cases and controls*** | ***Non-response rate*** |  |
| Degboé, Yannick et al.2022 | * |  |  | * | * | * | * |  | 5 |
| Nas, Kemal et al.2015 | * |  | * | * | * | * | * |  | 6 |
