# Supplementary material for: Epidemiology of Diabetes Mellitus in Patients With Psoriatic Arthritis: Current Evidence
Source: Diabetes Metab Res Rev. 2026 Oct 27;42(7):e70239. doi: 10.1002/dmrr.70239 (PMC13711012; doi:10.1002/dmrr.70239)
Supplement: Supplementary file 6 [file DMRR-42-e70239-s006.docx]

| **Table S8. Quality assessment of the non-treatment-disclosed cohort and case-control studies using the Newcastle-Ottawa Scale** | | | | | | | | | |
| --- | --- | --- | --- | --- | --- | --- | --- | --- | --- |
| **1. Cohort studies** | | | | | | | | | |
| ***Studies*** | ***Selection*** | | | | ***Comparability*** | ***Outcome*** | | | ***Total score*** |
|  | ***Representativeness of the exposed cohort*** | ***Selection of the non-exposed cohort*** | ***Ascertainment of exposure*** | ***Demonstration that the outcome of interest was not present at the start of study*** | ***Comparability of cohorts on the basis of the design or analysis*** | ***Assessment of outcome*** | ***Follow-up long enough for outcomes to occur*** | ***Adequacy of follow-up of cohorts*** |  |
| Ahmed, Nadeem et al.2016 | * | * | * | * | * | * |  |  | 6 |
| Alrubaiaan, Mishari T et al.2023 | * |  | * | * |  | * |  |  | 4 |
| Charlton, Rachel et al.2019 | * | * | * | * | * | * | * | * | 8 |
| Edson-Heredia, E et al.2015 | * | * | * | * | * | * | * |  | 7 |
| Jafri, Kashif et al.2017 | * |  | * | * | * | * | * | * | 7 |
| Kaine, Jeffrey et al.2019 | * |  | * | * | * | * | * | * | 7 |
| Radner, Helga et al.2017 | * | * | * | * | * | * | * | * | 8 |
| Koppikar, Sahil et al.2025 | * | * | * | * | * | * | * | * | 8 |
|  |  |  |  |  |  |  |  |  |  |
| **2. Case-control studies** | | | | | | | | | |
| ***Studies*** | ***Selection*** | | | | ***Comparability*** | ***Exposure*** | | | ***Total score*** |
|  | ***Is the case definition adequate*** | ***Representativeness of the cases*** | ***Selection of controls*** | ***Definition of controls*** | ***Comparability of cases and controls on the basis of the design or analysis*** | ***Ascertainment of exposure*** | ***Same method of ascertainment for cases and controls*** | ***Non-response rate*** |  |
| Hadi, Y et al.2022 | * |  | * | * | * | * | * |  | 6 |
| AlJohani, RoaA et al.2018 (1) | * | * |  |  | * | * | * | * | 6 |
| Kibari, Adi et al.2019 | * | * | * | * | * | * | * | * | 8 |
