# Supplementary material for: Epidemiology of Diabetes Mellitus in Patients With Psoriatic Arthritis: Current Evidence
Source: Diabetes Metab Res Rev. 2026 Oct 27;42(7):e70239. doi: 10.1002/dmrr.70239 (PMC13711012; doi:10.1002/dmrr.70239)
Supplement: Supplementary file 7 [file DMRR-42-e70239-s007.docx]

| **Table S7. Risk of bias of the non-treatment-disclosed cross-sectional studies assessed using the Agency for Healthcare Research and Quality tool** | | | | | | | | | | | | |
| --- | --- | --- | --- | --- | --- | --- | --- | --- | --- | --- | --- | --- |
| ***Studies*** | ***Q1*** | ***Q2*** | ***Q3*** | ***Q4*** | ***Q5*** | ***Q6*** | ***Q7*** | ***Q8*** | ***Q9*** | ***Q10*** | ***Q11*** | ***Scores*** |
| Atzeni, Fabiola et al.2023 | Y | N | Y | U | N | N | N | N | U | Y | Y | 4 |
| Chandrashekara, Srikantiah et al.2022 | Y | Y | Y | U | N | N | Y | N | N | N | U | 4 |
| Dreiher, Jacob et al.2013 | Y | Y | N | Y | N | Y | Y | Y | N | Y | U | 7 |
| Edens, Cuoghi et al.2018 | Y | Y | Y | Y | N | Y | Y | Y | Y | U | U | 8 |
| Furer, Victoria et al.2020 | Y | Y | Y | U | N | Y | N | Y | U | U | U | 5 |
| Han, Chenglong et al.2006 | Y | Y | Y | Y | N | Y | U | Y | U | N | U | 6 |
| Haque, Naba et al.2016 | Y | Y | Y | N | N | Y | Y | Y | U | N | Y | 7 |
| Huang, Rui et al.2024 | Y | Y | Y | Y | N | U | Y | U | U | Y | U | 6 |
| Landgren, Anton Jonatan et al.2021 | Y | Y | Y | N | N | N | U | Y | U | Y | U | 5 |
| Mazzillo, Mara et al.2022 | Y | Y | Y | U | N | Y | Y | Y | U | N | U | 6 |
| Skornicki, Michelle et al.2021 | Y | Y | Y | Y | N | N | Y | Y | U | Y | Y | 8 |
| Urbancek, Slavo et al.2016 | Y | Y | Y | Y | N | Y | Y | U | Y | N | U | 7 |
| Vega-Morales, David et al. 2024 | Y | Y | Y | U | N | U | Y | Y | U | Y | U | 6 |
| Chandrashekara, S et al.2025 | Y | Y | Y | U | N | U | Y | Y | N | N | U | 5 |
| Li, Jiashuai et al.2024 | Y | N | Y | U | N | Y | U | Y | N | Y | N | 5 |
| Puig, Luis et al.2013 | Y | Y | U | N | Y | Y | Y | Y | Y | Y | Y | 9 |
| Questions (Q):  Q1 Define the source of information (survey, record review)  Q2 List inclusion and exclusion criteria for exposed and unexposed subjects (cases and controls) or refer to previous publications  Q3 Indicate time period used for identifying patients  Q4 Indicate whether or not subjects were consecutive if not population-based  Q5 Indicate if evaluators of subjective components of study were masked to other aspects of the status of the participants  Q6 Describe any assessments undertaken for quality assurance purposes (e.g., test/retest of primary outcome measurements)  Q7 Explain any patient exclusions from analysis  Q8 Describe how confounding was assessed and/or controlled  Q9 If applicable, explain how missing data were handled in the analysis  Q10 Summarize patient response rates and completeness of data collection  Q11 Clarify what follow-up, if any, was expected and the percentage of patients for which incomplete data or follow-up was obtained | | | | | | | | | | | | |
| Y, Yes; N, No; U, Unclear | | | | | | | | | | | | |
