# Supplementary material for: Epidemiology of Diabetes Mellitus in Patients With Psoriatic Arthritis: Current Evidence
Source: Diabetes Metab Res Rev. 2026 Oct 27;42(7):e70239. doi: 10.1002/dmrr.70239 (PMC13711012; doi:10.1002/dmrr.70239)
Supplement: Supplementary file 8 [file DMRR-42-e70239-s008.docx]

Supplementary Figure Legends

Figure S1. **Prevalence of diabetes mellitus and sensitivity analyses in patients with psoriatic arthritis without treatment disclosure.** (A) Forest plot of the overall prevalence of DM. (B–E) Forest plots of prevalence stratified by age group (B), DM type (C), country (D), and continent (E). (F) Geographic distribution of pooled prevalence by continent. (G) Forest plot of prevalence stratified by study design. (H–K) Leave-one-out sensitivity analyses for the overall population (H), adults (I), European populations (J), and unclassified DM (K). In forest plots, squares represent study-specific prevalence estimates, horizontal lines indicate 95% confidence intervals, and diamonds represent pooled estimates. Sensitivity analyses show the pooled estimate after sequential omission of each study. DM, diabetes mellitus; CI, confidence interval.

Figure S2**. Assessment of small-study effects in patients with psoriatic arthritis without treatment disclosure.** Panels show the overall analysis (A-C), adults (D-F), European populations (G-I), and unclassified DM (J-L). Within each set of three panels, the left panel shows the funnel plot, the middle panel shows the trim-and-fill funnel plot, and the right panel shows the Egger regression plot. DM, diabetes mellitus; PsA, psoriatic arthritis.

Figure S3. **Prevalence of diabetes mellitus in patients with psoriatic arthritis with treatment disclosure.** (A) Forest plot of the overall prevalence of DM. (B–E) Forest plots of prevalence stratified by age group (B), DM type (C), continent (D), and country (E). (F) Geographic distribution of pooled prevalence by continent. (G, H) Forest plots of prevalence stratified by study design (G) and reported therapy category (H). Squares represent study-specific prevalence estimates, horizontal lines indicate 95% confidence intervals, and diamonds represent pooled estimates. DM, diabetes mellitus; CI, confidence interval.

Figure S4. **Sensitivity analyses of diabetes mellitus prevalence in patients with psoriatic arthritis with treatment disclosure**. (A–I) Leave-one-out sensitivity analyses for the overall population (A), adults (B), Asian populations (C), European adult populations (D), North American populations (E), cross-sectional studies (F), T2DM (G), unclassified DM (H), and the systemic therapy + biologics category (I). Each analysis shows the pooled prevalence estimate and its 95% confidence interval after sequential omission of each study. DM, diabetes mellitus; T2DM, type 2 diabetes mellitus; CI, confidence interval.

Figure S5. **Assessment of small-study effects in patients with psoriatic arthritis with treatment disclosure.** Panels show the overall analysis (A-C), adults (D-F), Asian populations (G-I), European populations (J-L), North American populations (M-O), cross-sectional studies (P-R), unclassified DM (S-U), T2DM in adults (V-X), and the systemic therapy + biologics category (Y-AA). Within each set of three panels, the left panel shows the funnel plot, the middle panel shows the trim-and-fill funnel plot, and the right panel shows the Egger regression plot. DM, diabetes mellitus; T2DM, type 2 diabetes mellitus; PsA, psoriatic arthritis.

Supplementary Table Legends

Table S1. PRISMA Checklist

Table S2. Moose checklist

Table S3. Characteristics of the included studies without treatment disclosure

Table S4. Characteristics of the included studies with treatment disclosure

Table S5. Risk of bias of the treatment-disclosed cross-sectional studies assessed using the Agency for Healthcare Research and Quality tool

Table S6. Quality assessment of the treatment-disclosed cohort and case-control studies using the Newcastle-Ottawa Scale

Table S7. Risk of bias of the non-treatment-disclosed cross-sectional studies assessed using the Agency for Healthcare Research and Quality tool

Table S8. Quality assessment of the non-treatment-disclosed cohort and case-control studies using the Newcastle-Ottawa Scale
