# Supplementary material for: Epidemiology of Diabetes Mellitus in Patients With Psoriatic Arthritis: Current Evidence
Source: Diabetes Metab Res Rev. 2026 Oct 27;42(7):e70239. doi: 10.1002/dmrr.70239 (PMC13711012; doi:10.1002/dmrr.70239)
Supplement: Supplementary file 9 [file DMRR-42-e70239-s009.docx]

**Table S4. Characteristics of the included studies with treatment disclosure**

| **Author- publication year** | **Study year** | **Study type** | **Region** | **F/M** | **Age[Mean±SD]** | **DM type** | **PsA(n)** | **Comorbidities (n)** | **Treatment method** | **Topical therapy** | **Systemic therapy** | **Biologics** | **Phototherapy** |
| --- | --- | --- | --- | --- | --- | --- | --- | --- | --- | --- | --- | --- | --- |
| Alonso, Sara et al.2016 (1) | 2011-2016 | Cohort study | Spain | 112/93 | 29 ± 7.2 | DM | 82 | 4 | Systemic therapy + Biologics | - | NSAIDs; GCs; MTX | Biologics | - |
| Alonso, Sara et al.2016 (2) | 2011-2016 | Cohort study | Spain | 112/93 | 52.8 ± 8.6 | DM | 123 | 21 | Systemic therapy + Biologics | - | NSAIDs; GCs; MTX | Biologics | - |
| Ciaffi, Jacopo et al.2022 (1) | 2019-2020 | Cross-sectional study | Italy | 195/213 | 57±14 | T1DM | 408 | 2 | Systemic therapy + Biologics | - | MTX; NSAIDs; LEF; SSZ; APR | ADA; CCs; CZP; ETN; GOL; SEC; TNFi; UST | - |
| Ciaffi, Jacopo et al.2022 (2) | 2019-2020 | Cross-sectional study | Italy | 195/213 | 57±14 | T2DM | 408 | 32 | Systemic therapy + Biologics | - | MTX; NSAIDs; LEF; SSZ; APR | ADA; CCs; CZP; ETN; GOL; SEC; TNFi; UST | - |
| Degboe, Yannick et al.2022 | 2016-2017 | Case-control study | France | 107/100 | 54.7±11.4 | DM | 207 | 25 | Systemic therapy + Biologics | - | NSAIDs; DMARDs; Steroids | bDMARDS | - |
| Eder, Lihi et al.2015 | 2010-2013 | Cohort study | Toronto | 133/105 |  | DM | 235 | 32 | Systemic therapy + Biologics | - | NSAIDS; DMARDs | Biologics | - |
| Eder, Lihi et al.2016 | 1978-2013 | Cohort study | Toronto | 612/479 | 43.8±12.8 | DM | 1091 | 59 | Systemic therapy + Biologics | - | DMARDs; CCs; NSAIDs | Anti-TNF biologics | - |
| Favarato, Maria Helena et al.2014 |  | Cohort study | Brazil | 79/74 |  | DM | 155 | 36/39? | Systemic therapy + Biologics | - | GCs；NSAIDs; Sulphassalazine; MTX; LEF | Anti-TNF biologics | - |
| Goh, Shiau Fui et al.2024 | 2007-2018 | Cross-sectional study | Malaysia | 1357/1399 |  | DM | 2756 | 563 | Systemic therapy + Biologics + Phototherapy | - | Acitretin; MTX; CCs; CsA; Hydroxyurea | Biologics | Phototherapy |
| Ishchenko, Alla et al.2024 | 2017-2020 | Cohort study | Belgium | 47/20 | 47.9±14.3 | T2DM | 67 | 8 | Topical therapy + Systemic therapy | NSAIDs | NSAIDs; GCs |  | - |
| Kamiya, Koji et al.2023 | 2017-2020 | Cross-sectional study | Japan | 1032/609 | 51.4±13.6 | DM | 1641 | 125/48 | Topical therapy + Systemic therapy + Biologics + Phototherapy | CCs; Vitamin D3; Tacrolimus | Oral medication; Etretinat; MTX; CsA; APR; CCs; NSAIDs | INF; ADA; CZP; UST; SEC; IXE; Brodalumab; Guselkuma; Risankizumab; Tildrakizumab; Biosimilar | PUVA;  NB‐UVB; BB‐UVB; Targeted UVB |
| Labitigan, Monalyn et al.2014 | 2001-2010 | Cross-sectional study | AMERICA | 159/135 | 55.7±11.9 | DM | 294 | 15 | Systemic therapy + Biologics | - | PDN; MTX; Any non-bDMARDS | Anti-TNF-α biologics | - |
| Nas, Kemal et al.2015 |  | Case-control study | Turkey | 75/98 | 41.8 | DM | 173 | 18 | Topical therapy | - | CCs |  | - |
| Ong, Ping Seung et al.2023 (1) | 2015-2022 | Cohort study | Malaysia | 48/51 | 56.5±12.4 | DM | 99 | 44 | Systemic therapy + Biologics | - | MTX; SSZ; LEF; Steroid; NSAIDs | TNFi; IL -17 A inhibitor; IL 23p19 inhibitor | - |
| Ong, Ping Seung et al.2023 (2) | 2015-2022 | Cohort study | Malaysia | 60/87 | 55.7±14.2 | DM | 148 | 40 | Systemic therapy + Biologics | - | MTX; SSZ; LEF; Steroid; NSAIDs | tumour necrosis factor; Interleukin -17 A inhibitor and Interleukin 23p20 inhibitor | - |
| Papadavid, E et al.2016 | 2011-2013 | Cross-sectional study | Greece | 42/43 | 53.63±14.49 | Type II DM | 85 | 17 | - | - |  |  | - |
| Pina Vegas, Laura et al.2021 (1) | 2010-2018 | Cohort study | France | 29010/34588 | 59.9±14.4 | DM | 63598 | 7707 | Topical therapy + Systemic therapy + Biologics | Topical therapies | csDMARDs (MTX; LEF; SSZ); NSAIDs; GCs; APR | biological originator and biosimilar DMARDs b/bsDMARDs(ETN; INF; ADA; CZP; GOL; Ustekizumab; SEC) | - |
| Pina Vegas, Laura et al.2021 (2) | 2010-2018 | Cohort study | France | 4603/3708 | 49.1±12.9 | DM | 8311 | 802 | Topical therapy + Biologics | Topical therapies |  | b/bsDMARDs (ETN; INF; ADA; CZP; GOL; Ustekizumab; SEC) | - |
| Pina Vegas, Laura et al.2021 (3) | 2010-2018 | Cohort study | France | 710/819 | 55.1±12.5 | DM | 1529 | 210 | Topical therapy + Systemic therapy | Topical therapies | APR |  | - |
| Pina Vegas, Laura et al.2021 (4) | 2010-2018 | Cohort study | France | 3781/5185 | 54.2±13.8 | DM | 8966 | 1004 | Topical therapy + Systemic therapy | Topical therapies | csDMARDs (MTX; LEF; SSZ) |  | - |
| Pina Vegas, Laura et al.2021 (5) | 2010-2018 | Cohort study | France | 11472/17995 | 59.0±14.8 | DM | 29467 | 4030 | Topical therapy + Systemic therapy | Topical therapies | NSAIDs; PDN |  | - |
| Polat Ekinci, Algun et al.2021 | 2020 | Cross-sectional study | Turkey | 98/35 | 44.6±13.5 | DM | 133 | 22 | Systemic therapy + Biologics | - | Methylprednisolone; colchicine; MTX; LEF | ETN; INF; ADA; UST; SEC; IXE; CZP | - |
| Queiro, Ruben et al.2018 |  | Cross-sectional study | Spain | 190/150 | 55±13 | Type II DM | 340 | 20/27 | Systemic therapy + Biologics | - | NSAIDs; GCs; MTX | Biologics | - |
| Queiro, Ruben et al.2019 |  | Cross-sectional study | Spain | 190/150 | 55±13 | T2DM | 340 | 20/27 | Systemic therapy + Biologics | - | NSAIDs; GCs; MTX | Biologics | - |
| Ritchlin, Christopher T et al.2019 (1) | 2015-2016 | Cohort study | Global | 1272/1129 | 50.8±12.8 | T1DM | 2401 | 39 | Topical therapy + Systemic therapy + Biologics + Phototherapy | tacrolimus | Aon-biologic/MTX; CsA; Mycophenolate mofetil; AZA; CCs | UST; INF; ADA; ETN; all other biologics | PUVA; UVB |
| Ritchlin, Christopher T et al.2019 (2) | 2015-2016 | Cohort study | Global | 1272/1129 | 50.8±12.8 | T2DM | 2401 | 325 | Topical therapy + Systemic therapy + Biologics + Phototherapy | tacrolimus | Non-biologics; MTX; CsA; mycophenolate mofetil; AZA; CCs | UST; INF; ADA; ETN; all other biologics | PUVA; UVB |
| Ross, Yael et al.2023 (1) | 1999-2019 | Cross-sectional study | AMERICA | 460/910 |  | DM | 1370 | 420 | Systemic therapy + Biologics | - | DMARDs; NSAIDs; JAKi | TNFi; IL inhibitors; PDE4i | - |
| Ross, Yael et al.2023 (2) | 1999-2019 | Cross-sectional study | AMERICA | 9166/15474 | - | DM | 24640 | 5660 | Systemic therapy + Biologics | - | DMARDs; NSAIDs; JAKi | TNFi; IL inhibitors; PDE4i | - |
| Scriffignano, Silvia et al.2023 (1) | 2012-2014 | Cross-sectional study | Italy | 182/158 | 55.4±12.3 | T2DM | 340 | 48 | Topical therapy + Systemic therapy + Biologics | Intra-articular CCs ; Topical CCs | NSAIDs; oral CCs; csDMARDs; MTX; APR | Anti-TNF-α; Anti-IL 17; Anti-IL-12/23 | - |
| Scriffignano, Silvia et al.2023 (2) | 2012-2014 | Cross-sectional study | Belgium | 264/199 | 52.7±12.3 | T2DM | 463 | 35 | Topical therapy + Systemic therapy + Biologics | Intra-articular CCs; Topical CCs | NSAIDs; oral CCs; csDMARDs; MTX; APR | Anti-TNF-α; Anti-IL 17; Anti-IL-12/23; | - |
| Shah, Kamal et al.2017 (1) | 2008-2015 | Real-world study | AMERICA | - | - | T2DM | 94302 | 19037 | Systemic therapy + Biologics |  | csDMARDs; NSAIDs; Steroids; tsDMARDs (APR, TOF) | Biologics | - |
| Sinnathurai, Premarani et al.2018 | 2015 | Cohort study | Australia | 200/290 | 50.4±12.1 | DM | 490 | 63 | Systemic therapy + Biologics | - | MTX; Prednis(ol)one; LEF; SSZ; HCQ; CsA; Gold IM; AZA; Penicillamine | ADA; ETN; INF; GOL; CZP; Rituximab; Abatacept; Tocilizumab; Anakinra | - |
| Song, Zhibo et al.2021 (1) | 2008-2010 | Cross-sectional study | China | 159/141 | - | T2DM | 300 | 55 | Systemic therapy + Biologics | - | MTX; LEF; SSZ; CsA; HCQ; Iguratimod; AZA; NSAIDs; GCs; Thalidomide; Traditional Chinese medicine | Etanercep; INF; ADA; SEC | - |
| Song, Zhibo et al.2021 (2) | 2008-2010 | Cross-sectional study | China | - | - | T2DM | 49 | 2 | Systemic therapy + Biologics | - | MTX; LEF; SSZ; CsA; HCQ; Iguratimod; AZA; NSAIDs; GCs; Thalidomide; Traditional Chinese medicine | Etanercep; INF; ADA; SEC | - |
| Song, Zhibo et al.2021 (3) | 2008-2010 | Cross-sectional study | China | - | - | T2DM | 68 | 13 | Systemic therapy + Biologics | - | MTX; LEF; SSZ; CsA; HCQ; Iguratimod; AZA; NSAIDs; GCs; Thalidomide; Traditional Chinese medicine | Etanercep; INF; ADA; SEC | - |
| Souza, Cacilda S et al.2019 | 2014-2015 | Cross-sectional study | Brazil | 152/141 | 52.0±12.8 | DM | 293 | 91 | Topical therapy + Systemic therapy + Biologics + Phototherapy | Topical therapies | Systemic therapy | Biologics | Phototherapy |
| Tam, Lai-Shan et al.2008 |  | Cross-sectional study | China | 48/54 | 48.7±12.2 | DM | 102 | 19 | Systemic therapy + Biologics | - | NSAIDs; DMARDs; MTX; CCs; Salazopyrine | Anti-TNF-α biologics | - |
| Velez, Nicole F et al.2012 | 2003-2009 | Cross-sectional study | AMERICA | 53/54 | 48.9±14.38 | DM | 107 | 23 | Topical therapy + Systemic therapy + Biologics | Topical therapies | tsDMARDs | Biologics | - |
| Zisman, Devy et al.2012 | 1998-2006 | Cross-sectional study | Israel | 64/85 | 58.2±14.6 | DM | 149 | 19 | Systemic therapy + Biologics | - | NSAIDs; DMARDs; MTX; steroids; PDN; SSZ ; AZA; colchicine; HCQ; LEF; CsA; gold salts | Anti-TNF agents | - |
| Eder, Lihi et al.2018 | - | Cross-sectional study | North America(5 Canadian, 2 US and 1 Israeli) | 1200/1054 | 52±13.8 | Diabete mellitus | 1327 | 177 | Systemic therapy + Biologics | - | NSAIDs; DMARDs; CCs | Biologics | - |
| Tekin, Hasan Gokcer et al.2019 (1) | 2015 | Cross-sectional study | Denmark | 2333/3278 | 57.0±14.5 | DM | 5611 | 631 | Topical therapy + Systemic therapy | Topical therapies | Systemic therapy | - | - |
| Tekin, Hasan Gokcer et al.2019 (2) | 2015 | Cross-sectional study | Denmark | 1399/2080 | 57.9±13.6 | DM | 3479 | 426 | Topical therapy + Systemic therapy | Topical therapies | DMARDs | - | - |
| Tekin, Hasan Gokcer et al.2019 (3) | 2015 | Cross-sectional study | Denmark | 677/810 | 53.7±12.8 | DM | 1487 | 167 | Topical therapy + Systemic therapy + Biologics | Topical therapies | Systemic therapy | Biologics | - |
| Khraishi, Majed et al. 2014 (1) | 2008-2011 | Cross-sectional study | Canada | 40/44 | 48.04±10.55 | DM | 84 | 5/6 | Topical therapy + Systemic therapy + Biologics | Steroid cream, Calcipotriene | MTX; SSZ; HCQ; PDN | ETN; INF; ADA | - |
| Khraishi, Majed et al. 2014 (2) | 2008-2011 | Cross-sectional study | Canada | 58/54 | 49.69±10.34 | DM | 112 | 9/7 | Topical therapy + Systemic therapy + Biologics | Steroid cream, Calcipotriene | NSAID，MTX; SSZ; HCQ; Plaquenil; PDN | ETN; INF; ADA | - |
| Khraishi, Majed et al.2011 (1) | 2008-2010 | Cross-sectional study | Canada | 22/35 | 41.64±14.41 | DM | 57 | 2/5 | Topical therapy + Systemic therapy + Biologics | Steroid cream | NSAID，MTX; SSZ; HCQ; Plaquenil; PDN | ETN; INF; ADA; GOL | - |
| Khraishi, Majed et al.2011 (2) | 2008-2010 | Cross-sectional study | Canada | 62/46 | 34.66±12.60 | DM | 108 | 6/6 | Topical therapy + Systemic therapy + Biologics | Steroid cream | NSAID，MTX; SSZ; HCQ; Plaquenil; PDN | ETN; INF; ADA; GOL | - |
| Alp, Gulay et al.2025 | 2021-2023 | Cohort study | Turkey | 55/116 | 48.16±11.23 | DM | 171 | 30 | Systemic therapy + Biologics | - | MTX; LEF; SSZ; GCs | TNFi; SEC; UST; IXE; Guselkumab | - |
| Freites-Nunez, Dalifer et al.2024 | 20007-2022 | Cohort study | Spain | 67/74 | 48.1±13.2 | DM | 141 | 7 | Systemic therapy + Biologics | - | Monotherapy; MTX; LEF; SSZ; AZA; JAKi (TOF, Baricitinib, Upadacitinib) | TNFi (INF, ADA, ETN; CZP, GOL); CTLA4–Ig (Abatacept); anti-IL-17 (IXE, SEC); anti-IL-12/23 (UST); anti-IL-23 (Guselkumab) | - |
| Kharouf, Fadi et al.2024 | 1978-2023 | Cohort study | Canada | 746/590 | 44.9±13.1 | DM | 1336 | 95 | Systemic therapy + Biologics | - | tsDMARDs (APR, TOF, upadacitinib); csDMARDs (MTX, SSZ, LEF) | TNFi; IL-17i; IL-23i (including IL-12/IL-23i) | - |
| Guła, Zofia et al.2024 | - | Cross-sectional study | Poland | 121/146 | 48.8±13.0 | DM | 267 | 21 | Systemic therapy + Biologics | - | NSAIDs; csDMARDs; tsDMARDs | bDMARDS | - |
| Pei, Wenwen et al.2025 | 2021-2022 | Cross-sectional study | China | - | - | DM | 91 | 19 | Systemic therapy + Biologics | - | NSAIDs; GCs; MTX; LEF; SSZ; Iguratimod; HCQ; JAKi | TNFi; anti-IL-17A monoclonal inhibitors | - |

**Abbreviation:** F/M, Female/Male; DM, Diabetes Mellitus; PsA, Psoriatic Arthritis; NSAIDs, Nonsteroidal Anti-Inflammatory Drugs; GCs, Glucocorticoids; MTX, Methotrexate; T1DM, Type 1 Diabetes Mellitus; LEF, Leflunomide; SSZ, Sulfasalazine; APR, Apremilast; ADA, Adalimumab; CCs, Corticosteroids; CZP, Certolizumab pegol; ETN, Etanercept; GOL, Golimumab; SEC, Secukinumab; TNFi, Tumor Necrosis Factor Inhibitors; UST, Ustekinumab; T2DM, Type 2 Diabetes Mellitus; DMARDs, Disease-Modifying Antirheumatic Drugs; bDMARDs, Biologic DMARDs; CsA, Cyclosporine A; INF, Infliximab; IXE, Ixekizumab; PUVA, Psoralen Plus Ultraviolet A; NB‐UVB, Narrowband Ultraviolet B; BB‐UVB, Broadband Ultraviolet B; PDN, Prednisone; IL, Interleukin; csDMARDs, conventional synthetic Disease-Modifying Antirheumatic Drugs; AZA, Azathioprine; JAKi, Janus Kinase Inhibitors; PDE4i, Phosphodiesterase 4 inhibitors; HCQ, Hydroxychloroquine; tsDMARDs, targeted synthetic DMARDs; TOF, Tofacitinib
